# Gamma frequency sensory stimulation in mild probable Alzheimer’s dementia patients: Results of feasibility and pilot studies

**DOI:** 10.1371/journal.pone.0278412

**Published:** 2022-12-01

**Authors:** Diane Chan, Ho-Jun Suk, Brennan L. Jackson, Noah P. Milman, Danielle Stark, Elizabeth B. Klerman, Erin Kitchener, Vanesa S. Fernandez Avalos, Gabrielle de Weck, Arit Banerjee, Sara D. Beach, Joel Blanchard, Colton Stearns, Aaron D. Boes, Brandt Uitermarkt, Phillip Gander, Matthew Howard, Eliezer J. Sternberg, Alfonso Nieto-Castanon, Sheeba Anteraper, Susan Whitfield-Gabrieli, Emery N. Brown, Edward S. Boyden, Bradford C. Dickerson, Li-Huei Tsai

**Affiliations:** 1 Picower Institute for Learning and Memory, Massachusetts Institute of Technology, Cambridge, Massachusetts, United States of America; 2 Department of Brain and Cognitive Sciences, Massachusetts Institute of Technology, Cambridge, Massachusetts, United States of America; 3 Department of Neurology, Massachusetts General Hospital, Boston, Massachusetts, United States of America; 4 Department of Neurology, Harvard Medical School, Boston, Massachusetts, United States of America; 5 McGovern Institute, Massachusetts Institute of Technology, Cambridge, Massachusetts, United States of America; 6 Health Sciences and Technology, Massachusetts Institute of Technology, Cambridge, Massachusetts, United States of America; 7 Department of Behavioral Neuroscience, Northeastern University, Boston, Massachusetts, United States of America; 8 Division of Sleep Medicine, Harvard Medical School, Boston, Massachusetts, United States of America; 9 Department of Computer Science, Stanford University, Stanford, California, United States of America; 10 Department of Pediatrics, Neurology, & Psychiatry, University of Iowa Hospitals and Clinics, Iowa City, Iowa, United States of America; 11 Department of Neurosurgery, University of Iowa Hospitals and Clinics, Iowa City, Iowa, United States of America; 12 Neuroscience Institute, University of Iowa, Iowa City, Iowa, United States of America; 13 Department of Neurology, Milford Regional Neurology, Milford, Massachusetts, United States of America; 14 Department of Neurology, University of Massachusetts Medical School, Worcester, Massachusetts, United States of America; 15 Institute for Medical Engineering and Sciences, Massachusetts Institute of Technology, Cambridge, Massachusetts, United States of America; 16 Institute for Data Systems and Society, Massachusetts Institute of Technology, Cambridge, Massachusetts, United States of America; 17 Department of Anesthesia, Critical Care and Pain Medicine, Massachusetts General Hospital, Boston, Massachusetts, United States of America; 18 Department of Biological Engineering, Massachusetts Institute of Technology, Cambridge, Massachusetts, United States of America; 19 Center for Neurobiological Engineering, Massachusetts Institute of Technology, Cambridge, Massachusetts, United States of America; 20 Koch Institute, Massachusetts Institute of Technology, Cambridge, Massachusetts, United States of America; 21 Howard Hughes Medical Institute, Cambridge, Massachusetts, United States of America; 22 Broad Institute of Harvard and Massachusetts Institute of Technology, Cambridge, Massachusetts, United States of America; University of Toronto, CANADA

## Abstract

Non-invasive Gamma ENtrainment Using Sensory stimulation (GENUS) at 40Hz reduces Alzheimer’s disease (AD) pathology such as amyloid and tau levels, prevents cerebral atrophy, and improves behavioral testing performance in mouse models of AD. Here, we report data from (1) a Phase 1 feasibility study (NCT04042922, ClinicalTrials.gov) in cognitively normal volunteers (n = 25), patients with mild AD dementia (n = 16), and patients with epilepsy who underwent intracranial electrode monitoring (n = 2) to assess safety and feasibility of a single brief GENUS session to induce entrainment and (2) a single-blinded, randomized, placebo-controlled Phase 2A pilot study (NCT04055376) in patients with mild probable AD dementia (n = 15) to assess safety, compliance, entrainment, and exploratory clinical outcomes after chronic daily 40Hz sensory stimulation for 3 months. Our Phase 1 study showed that 40Hz GENUS was safe and effectively induced entrainment in both cortical regions and other cortical and subcortical structures such as the hippocampus, amygdala, insula, and gyrus rectus. Our Phase 2A study demonstrated that chronic daily 40Hz light and sound GENUS was well-tolerated and that compliance was equally high in both the control and active groups, with participants equally inaccurate in guessing their group assignments prior to unblinding. Electroencephalography recordings show that our 40Hz GENUS device safely and effectively induced 40Hz entrainment in participants with mild AD dementia. After 3 months of daily stimulation, the group receiving 40Hz stimulation showed (i) lesser ventricular dilation and hippocampal atrophy, (ii) increased functional connectivity in the default mode network as well as with the medial visual network, (iii) better performance on the face-name association delayed recall test, and (iv) improved measures of daily activity rhythmicity compared to the control group. These results support further evaluation of GENUS in a pivotal clinical trial to evaluate its potential as a novel disease-modifying therapeutic for patients with AD.

## Introduction

Alzheimer’s disease (AD) is a multi-faceted neurodegenerative disorder characterized by excessive accumulation of amyloid-beta and hyper-phosphorylated tau neurofibrillary tangles as major pathological features [1]. In addition to this molecular pathology, disruptions in neuronal network oscillations are observed in AD [2–5]. For example, gamma frequency band (30–80 Hz) oscillations on electroencephalogram (EEG) recordings, which are related to cognitive functions such as attention and memory [6–8], are altered both in human patients with AD [9–14] and mouse models of the disease [15–17]. Increasing the amplitude of gamma band oscillations through genetic modifications or optogenetic stimulation can reduce amyloid levels and improve memory in AD model mice [15, 18, 19]. However, the relationship between AD pathology and disruptions in amplitude or synchronization of gamma band oscillations is not yet fully elucidated.

Recently, we discovered that non-invasive entrainment of gamma frequency oscillations using light flickering at 40Hz (Gamma ENtrainment Using Sensory stimuli, GENUS) reduced amyloid load and induced glial response in the visual cortex of AD model mice, effectively attenuating AD-related pathology [17]. Entrained gamma frequency oscillations were observed across multiple brain regions beyond the visual cortex, and areas of the brain with entrainment showed preserved neuronal and synaptic densities [20]. Furthermore, mouse models of AD showed improved cognitive performance after daily stimulation with 40Hz over a period of 3–6 weeks (chronic GENUS) [20]. In another study, we found that auditory stimulation at 40Hz also led to gamma frequency oscillation entrainment and ameliorated pathology and that combined 40Hz visual and auditory stimulation produced enhanced beneficial effects in AD model mice [21]. These positive effects of gamma frequency oscillation entrainment using GENUS in mouse models suggest that it is worth pursuing GENUS as a non-invasive therapeutic avenue for the treatment of AD in humans. We hypothesize that inducing 40Hz entrainment in patients with mild probable AD dementia may modify pathophysiology related to neurodegeneration, leading to improved memory and function.

Previous studies investigating the use of sensory stimulation to induce entrainment showed that 40Hz auditory stimuli created the highest response as measured by both EEG and increased regional cerebral blood flow in healthy participants [22]. A frequency of 40 Hz was also chosen for other neuromodulation modalities such as transcranial alternative current (TACS) in studies targeted at improving abstract reasoning, working memory, and insight in cognitively normal volunteers [23–25]. Based on these studies and on data on the effect of different gamma frequencies in mouse models of AD, we concluded that 40Hz was the optimal frequency to evaluate whether induced gamma entrainment can prevent neurodegeneration and improve cognition [17, 20, 21]. We opted to use combined visual and auditory stimulation based on our data in AD mouse models (21) and reports that simultaneous stimuli with multiple sensory modalities can produce responses that combine the effect of each of the constituent uni-sensory stimuli [26–29].

A recent open-label study administered 4 or 8 weeks of sensory stimulation in 10 patients with mild cognitive impairment and demonstrated entrainment by EEG and increased functional connectivity as measured by functional MRI after 8 weeks of stimulation [30]. In a small randomized, placebo-controlled study of 6 months of 40Hz sensory stimulation in patients with mild to moderate AD dementia, the active group exhibited improved sleep and no decline in activities of daily living [31]. Early evidence thus suggests that 40Hz stimulation is well-tolerated, engages the target brain regions, and induces potential benefits. Here, we evaluate whether GENUS-induced gamma entrainment can be safely applied in patients with mild probably AD dementia to affect neural networks and possibly slow neurodegeneration and improve cognition.

We report two studies of 40Hz combined visual and auditory GENUS: The first study (Phase 1 study) was designed to assess if a single brief GENUS treatment could safely produce 40 Hz entrainment in cognitively normal younger or older volunteers (n = 27), patients with mild AD dementia (n = 16), and patients with epilepsy who were undergoing intracranial electroencephalographic monitoring (n = 2). The second study (Phase 2A study) was a single-blinded, randomized, placebo-controlled pilot trial in patients with mild probable AD dementia (n = 15) designed to assess safety, compliance, entrainment, and exploratory clinical outcomes after daily 1-hour 40Hz sensory stimulation for 3 months. The main objective of this paper is to report on the feasibility and safety of daily, at-home light and sound GENUS in patients with mild probable AD dementia and to characterize 40Hz entrainment in cognitively normal adults and patients with AD dementia. Exploratory objectives included the effects of daily GENUS on cognition, sleep, and AD biomarkers such as structural and functional MRI. The Phase 2A study was not statistically powered to detect differences between the control and active groups on these exploratory measures.

## Materials and methods

### Study design

The Phase 1 study (NCT04042922, ClinicalTrials.gov) was performed at the Massachusetts Institute of Technology (MIT) and the University of Iowa. The Phase 2A study (NCT04055376) was performed at MIT. The Phase 2A study and the part of the Phase 1 study conducted at MIT were approved by the Committee on the Use of Humans as Experimental Participants (COUHES) at MIT. The part of the Phase 1 study conducted at the University of Iowa was approved by the University of Iowa Institutional Review Board. These studies were carried out in accordance with the Code of Ethics of the World Medical Association. Written consent was obtained by study personnel from all participants; for the AD cohort, caregivers also gave written consent to participate in these studies.

The Phase 1 study involved one study visit, during which the participant underwent EEG recording with sensory stimulation. The primary outcome measures for this study were safety and degree and localization of entrainment.

**Fig 1:**
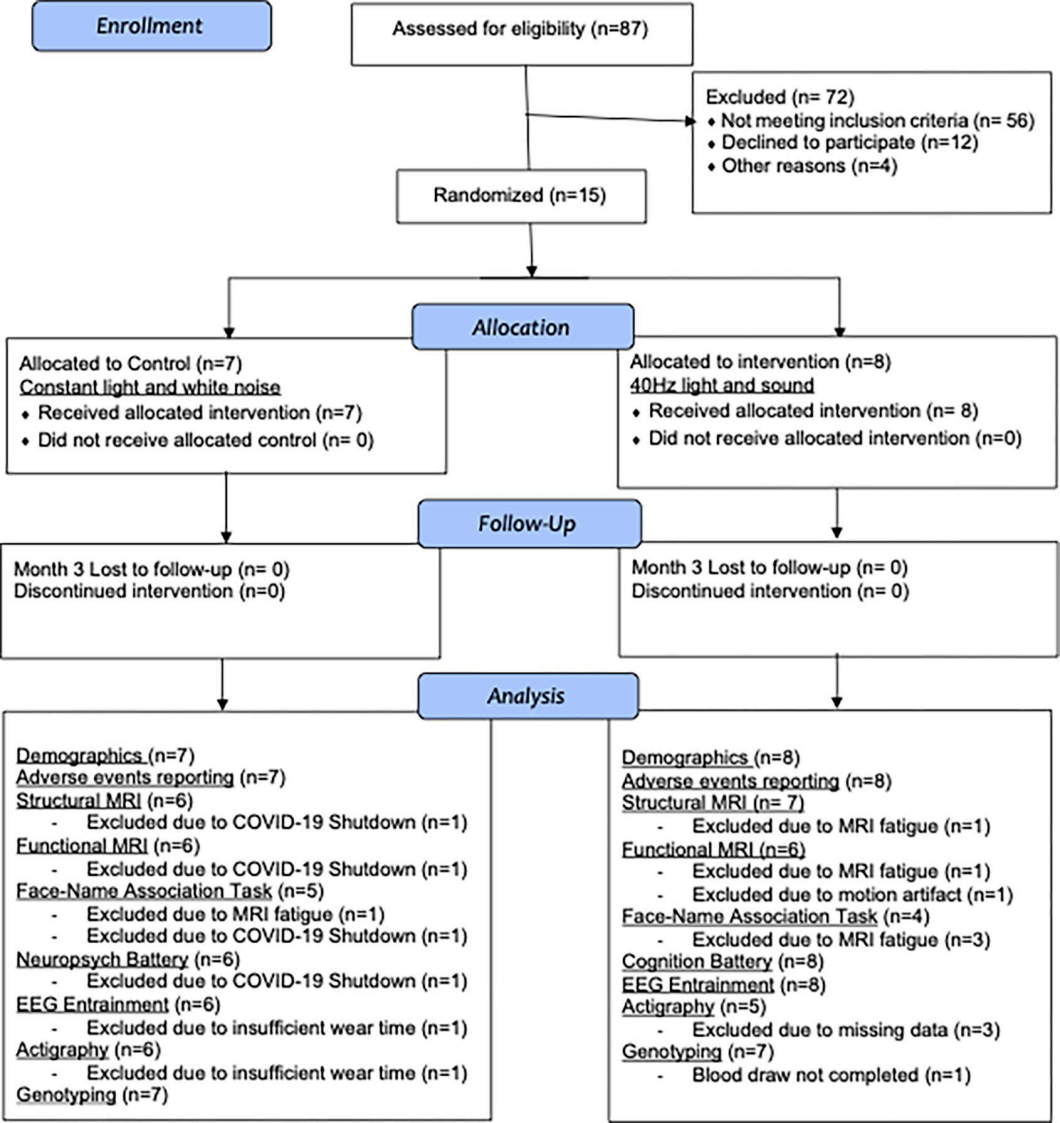
CONSORT flow diagram: Phase 2A study cohort. Participant recruitment, allocation, follow-up, and analysis counts by activity.

The Phase 2A study was a single-blinded, randomized, placebo-controlled study in which participants were given stimulation devices to be used for 1 hour daily for 6 months (S1 Fig). Participants were randomized 1:1 to control (constant light and white noise) and active groups (40 Hz light and sound). Participants and their caregivers were blinded to the randomization assignments. Participants underwent baseline assessments including a neuropsychological test battery, EEG with sensory stimulation, magnetic resonance imaging (MRI), and blood collection for sequencing and wore actigraphy devices throughout the study. The MRI protocol included a T1-weighted structural sequence and functional MRI sequences at rest and during the performance of a memory task. While the study protocol called for a repeat of the neuropsychological test battery, EEG with sensory stimulation, and MRI at 3 and at 6 months, the 6-month visit could not be completed due to the COVID-19 pandemic. Therefore, we report here only the results up to 4 months, including the 3-month but not the 6-month timepoint (see Fig 1 for a CONSORT diagram for the study). The primary outcome measures for this study were safety and compliance. Safety was assessed with adverse events questionnaires completed over weekly phone calls with participants and with evaluations for aberrant ictal spikes in response to stimulation using EEG at baseline and 3 months. Compliance was assessed using the built-in time-stamp log of when the device was used and through a mounted camera that took pictures every 5 seconds when the device was turned on. Exploratory outcomes included effects on cognition, based on performance on the neuropsychological test battery; changes in structural and functional MRI; and activity, based on actigraphy devices worn by participants throughout the study. Investigators, study site staff, patients, and caregivers were initially blinded to the randomization assignment, except for those investigators who prepared the GENUS devices, until all data were cleaned and primary analyses were complete. At that point, each participant was asked to state the group to which they thought they had been randomized (active or control).

### Participants

The Phase 1 study involved three cohorts recruited at MIT (demographics in Table 1) and one cohort recruited at the University of Iowa. The MIT cohorts consisted of (i) cognitively normal adults aged 18–35 years (young group, n = 13); (ii) cognitively normal adults aged 50–100 years (older group, n = 12); and (iii) older adults aged 50–100 years with a diagnosis of probable AD dementia and a Mini-Mental State Examination (MMSE) [32] score of 19–26 at screening (mild AD group, n = 16). The primary exclusion criteria were active treatment with N-methyl-D-aspartate (NMDA) receptor antagonist (e.g., memantine), anti-epileptic agent, or psychiatric agent (e.g., antidepressant, antipsychotic) and history of seizure or stroke within 24 months prior to the study participation. Full inclusion and exclusion criteria are listed in S1 Table. Following cognitive assessment, participants’ EEG was recorded during 40Hz GENUS stimulation.

**Table 1:** Demographics and baseline clinical characteristics of Phase 1 study participants at MIT.

Characteristic		Young cognitively normal (n = 13)	Older cognitively normal (n = 12)	Mild AD (n = 16)
**Years of age** (mean, sd)		25.6 (3.9)	64.9 (6.3)	75.8 (7.9)
**Female sex** (n, %)		6 (46)	6 (50)	9 (56)
**Years of education** (mean, sd)		17.1 (1.7)	17.8 (1.8)	15.2 (4.0)
**MMSE^a^** (median, range)		30 (28–30)	30 (28–30)	22 (19–25)
**MoCA^a^** (median, range)		N/A	N/A	18 (12–26)
**Global CDR^b^** (n, %)	1.0	N/A	N/A	12 (75)
	0.5	N/A	N/A	4 (25)
**Reported race: white** (n, %)		7 (54)	11 (92)	13 (81)

Percentages are rounded to the nearest integer. Abbreviations: AD, Alzheimer’s Disease; CDR, Clinical Dementia Rating; MMSE, Mini Mental State Examination; MoCA, Montreal Cognitive Assessment.

^a^ Ranges from 0 to 30, with a higher score indicating less impairment.

^b^ Ranges from 0 to 3, where 0 = normal, 0.5 = very mild dementia, 1 = mild dementia, 2 = moderate dementia, 3 = severe dementia.

The University of Iowa cohort consisted of two neurosurgical patients with medically intractable epilepsy recruited at the University of Iowa Hospitals & Clinics. These patients were included in the study because their intracranial electrodes allowed monitoring of subcortical brain regions for 40Hz entrainment. Both patients were male, aged 19 (patient 483) and 35 (patient 493), and admitted to the hospital for 7–14 days for invasive monitoring with intracranial electrodes as part of their epilepsy treatment plan. After the final surgical treatment plan was agreed upon between the clinical team and the patient, 1–2 days before the planned resection and after the patient had restarted anti-epileptic medications, the patient’s intracranial EEG was recorded during 40Hz GENUS stimulation.

The Phase 2A study included mild probable AD patients who met the National Institute on Aging and Alzheimer’s Association diagnostic criteria for mild probable AD dementia [33]. Participants in the “mild AD” cohort of the Phase 1 study who satisfied these clinical criteria were offered participation in the Phase 2A study, and 15 of the mild AD participants from the Phase 1 study did enroll in the Phase 2A study. The participants in the Phase 2A study are therefore a subgroup of the participants in the mild AD cohort of the Phase 1 study. The diagnosis was confirmed by the investigator (DC), a board-certified neurologist with subspecialty training in memory disorders, based on interviews with the patient and their caregiver along with neurological assessments. The primary exclusion criteria included an MMSE score outside of 19–26 at screening and a history of seizure. A full list of inclusion and exclusion criteria is given in S1 Table, mild AD group. Participant demographics for the Phase 2A study are listed in Table 2.

**Table 2:** Demographics and baseline clinical characteristics of Phase 2A study participants (AD patients).

Characteristic		Control (n = 7)	Active (n = 8)	Inter-group difference (p-value)
**Years of age** (mean, sd)		71.2 (8.2)	77.6 (7.5)	0.17
**Female sex** (n, %)		5 (71)	5 (63)	0.74
**Years of education** (mean, sd)		12.4 (4.2)	17.8 (2.1)	0.01*
**MMSE^a^** (median, range)		22.0 (18.0–24.0)	23.5 (19.0–27.0)	0.32
**MoCA^a^** (median, range)		15.0 (7.0–20.0)	19.5 (14.0–23.0)	0.09
**ADAS-Cog^b^** (media, range)		19.0 (10.33–24.0)	15.0 (6.66–30.0)	0.26
**FNA-DRT** (median, range)		8.0 (6.0–10.0)^d^	5.5 (5.0–9.0)^e^	0.23
**Global CDR^c^ (n, %)**	1.0	6 (86)	5 (63)	0.31
	0.5	1 (14)	3 (37)	
**APOE4^f^ (n, %)**	Carriers	4 (57)	5 (71)	0.58
	Non-carriers	3 (43)	2 (29)	
**Reported race: white** (n, %)		5 (71)	8 (100)	0.1
**1^st^ degree family member with probable AD** (n, %)		1 (14)	4 (50)	0.14
**Taking Aricept** (n, %)		2 (29)	4 (50)	0.39

Percentages are rounded to the nearest integer. Abbreviations: ADAS-Cog, Alzheimer’s Disease Assessment Scale-Cognitive Subscale; APOE4, apolipoprotein E epsilon4 allele; CDR, Clinical Dementia Rating; FNA-DRT, Face-Name Association Delayed Recall Test; MMSE, Mini Mental State Examination; MoCA, Montreal Cognitive Assessment.

^a^ Ranges from 0 to 30, with a higher score indicating less impairment.

^b^ Ranges from 0 to 70, with a higher score indicating greater impairment.

^c^ Ranges from 0 to 3, where 0 = normal, 0.5 = very mild dementia, 1 = mild dementia, 2 = moderate dementia, 3 = severe dementia

^d^ n = 5

^e^ n = 4

^f^ n = 7 for active group

### Sensory stimulation

**Fig 2:**
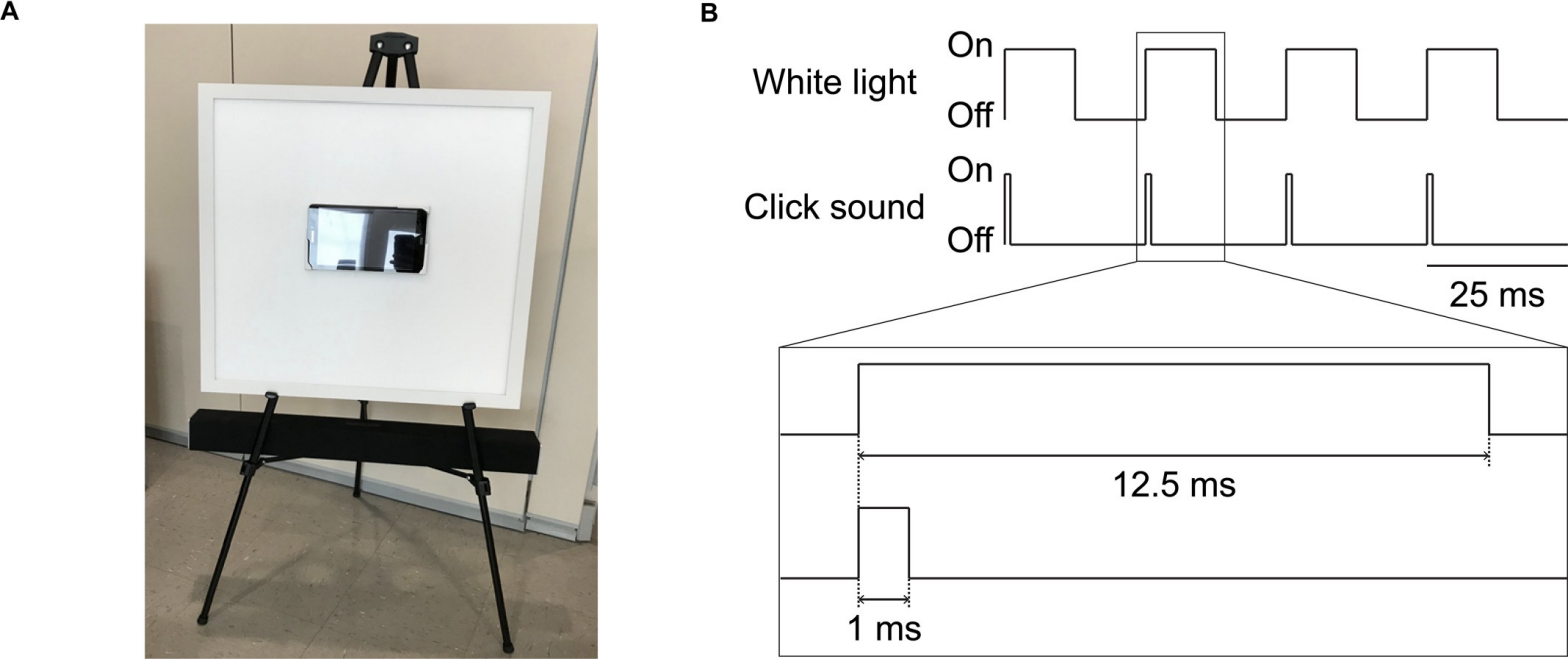
GENUS device and 40Hz stimulation schematic. (A) Light and sound stimulation device. The white board is a programmable LED light display panel that measures at 2 feet x 2 feet. Sitting at the center of the light panel is a tablet loaded with videos to keep the viewer’s attention on the device. The black bar below the light panel is the programmable soundbar audio system. The device is supported by the legs of a black easel and positioned so the tablet is eye level when the viewer is seated 5 feet away. (B) Schematic of the electrical signals that turn the white light and the click sound pulses on or off for concurrent light and sound stimulation at 40Hz.

Sensory stimulation was delivered using a white light LED panel (2’x2’, correlated color temperature 3900-4000K = natural white color) that was modified for flicker and brightness control (Neltner Labs, Boston, MA) and a sound system (SB2920-C6, VIZIO, Irvine, CA; or LP-2024A+, Lepy connected to BRS40, BOSS, Hamamatsu, Japan; Fig 2A). For the visual stimulation, both the control (constant white light) and active groups (40Hz light flicker; Fig 2B) received average light intensity around 390–400 lux at the participant’s eyes. For the auditory stimulation, the 40Hz sound was 78dB (cognitively normal group) or 68dB (patients with mild AD or epilepsy), with a 1ms rectangular pulse in each 25ms interval (4% duty cycle, 24ms isi; Fig 2B). The control sound was white noise at 68dB. The LED panel and the speaker were controlled by a custom circuit board that housed a Teensy USB Development Board (version 3.6, PJRC, Portland, OR).

### Evaluation of induced entrainment using scalp EEG recording with sensory stimulation

Participants receiving sensory stimulation were seated five feet from the device and had simultaneous EEG recording. Each participant was exposed to three 40Hz stimulation conditions (visual, auditory, and combined) and three control conditions (visual: constant white light, auditory: white noise, and combined), with each condition lasting for about 3 minutes (for cognitively normal participants) or 1 minute (for patients with mild AD), and to 1 minute of baseline recording between each condition. Baseline recordings were done with the device turned on but with the light obscured and the audio volume set at 0dB. The order of stimulation conditions was randomized for each participant, and each EEG session was captured in one continuous recording. For the combined stimulation conditions, auditory and visual pulses were temporally aligned at their onset (Fig 2B).

Scalp EEG was recorded throughout the stimulation experiments using an ActiveTwo system (BioSemi, Amsterdam, The Netherlands), with 32 scalp electrodes arranged according to the international 10–20 system. Electrooculogram (EOG) electrodes were used to monitor eye blinks and lateral eye movements, with one EOG electrode placed near the infraorbital ridge of the left eye and another electrode placed near the lateral canthus of the right eye. Two additional electrodes were placed over the mastoids bilaterally. The magnitude of the offset value was kept below 40 mV for each electrode. EEG signals were recorded with a low-pass hardware filter with a half-power cutoff at 104 Hz and then digitized at 512 Hz with 24 bits of resolution. “Trigger events” were manually inserted during the experiment to denote the start and finish of each stimulation period. EEG recordings were analyzed using the EEGLAB toolbox [34, 35] and custom scripts. Collected data were filtered (0.1–100 Hz bandpass), referenced to the average of the mastoid electrodes, and subjected to independent component analysis using the EEGLAB runica function. Components were manually reviewed and rejected if they represented eye blink, eye lateral movement, channel noise, or muscle artifacts. The pruned data were re-referenced using the surface Laplacian referencing scheme to remove volume conduction and eliminate artifacts from reference electrodes, increasing the sensitivity of each scalp electrode to local cortical sources [36]. We used a method similar to the Hjorth Laplacian method [37], in which the mean signal of the neighboring electrodes was subtracted from the signal at each electrode. For each of the 40Hz stimulation condition recordings, control condition recordings, and baseline recordings, the power spectral density (PSD) was calculated using the middle 30sec (for the scalp EEG data) or 45sec (for the intracranial EEG data) segment of the recording.

### Intracranial EEG recording with sensory stimulation

Each of the two epilepsy participants was exposed to three 40Hz stimulation conditions (visual, auditory, and combined), with each condition lasting for about 1 minute. Intracranial EEG was recorded throughout the stimulation experiments using electrode arrays (Ad-Tech Medical Instrument, Oak Creek, WI) that included stereotactically-implanted depth electrodes (4–8 macro contacts per electrode, spaced 5-10mm apart) and grid arrays (containing platinum-iridium disc contacts, with 2.3mm exposed diameter, 5-10mm inter-contact distance, embedded in a silicon membrane) placed on the cortical surface. A subgaleal electrode was used as a reference. Data acquisition was controlled by an ATLAS Neurophysiology system (Neuralynx, Bozeman, MT). Collected data were amplified, filtered (0.1-500Hz bandpass), digitized at 2kHz, and stored for subsequent offline analysis.

### Detection of epileptiform discharges

All EEG recordings were reviewed for the presence of epileptiform discharges by an expert neurophysiologist (EJS) blinded to the timing and the duration of the stimulation. For scalp EEG recordings, raw signals without any preprocessing were examined for epileptiform discharges. For intracranial EEG, raw signals were bandstop filtered between 59 and 61Hz due to strong line noise at 60Hz and bandpass filtered between 0.1 and 100Hz before examination.

### MRI

MRI data were acquired using a 3-Tesla Siemens Tim Trio scanner (Siemens, Erlangen, Germany) paired with a 12-channel phased-array whole-head coil. Head motion was restrained with foam pillows. Sequences included 3D T1-weighted magnetization prepared rapid acquisition gradient echo (MP-RAGE) anatomical images. Functional T2*-weighted images were acquired using a gradient-echo echo-planar pulse sequence sensitive to bold oxygenation level-dependent (BOLD) contrast [38, 39]. To allow for T1-equilibration effects, 4 dummy volumes were discarded prior to acquisition. Functional resting data were acquired while the participant was instructed to rest with eyes open for a period of 5 minutes consisting of 120 volumes. Functional task data were recorded during the visual presentation of faces paired with fictional names in a modified novel vs repeated design [40], for a total of 98 volumes per run. Online prospective acquisition correction (PACE) was applied to the EPI sequence. Structural MRI data was analyzed using FSL (https://fsl.fmrib.ox.ac.uk/fsl) and FreeSurfer (http://surfer.nmr.mgh.harvard.edu). Functional MRI data was analyzed using CONNToolbox [41] and Statistical Parametric Mapping (SPM - https://www.fil.ion.ucl.ac.uk/spm/).

### Face-name association delayed recall task (FNA-DRT)

Immediately following the last part of fMRI imaging, participants were tested on their delayed recall of a subset of the face-name pairs that had been presented to them during the fMRI. A subset of participants was unable to complete the task due to the duration of the MRI session or because they missed the entire MRI scan during the Month 3 visit and were removed from this analysis (active n = 4, control n = 5).

### Actigraphy

Activity was assessed using an actigraphy device (ActiGraph Link GT9X+, Firmware 1.7.2, ActiGraph, Pensacola, FL) worn on the non-dominant wrist continuously throughout the study. Five 7-day recordings were analyzed, starting: (i) with at home installation of the stimulation device (baseline), (ii) after 30 days (Month 1), (iii) after 60 days (Month 2), (iv) after a median of 101 days (range: 85–109 days, Month 3), and (v) after 120±15 days (Month 4). Data were visually inspected to confirm that recordings were present followed by Wear Time Validation preprocessing (ActiLife software version 6.13.4, ActiGraph, Pensacola, FL) using a 90 minute non-wear threshold [42, 43]. Participants who had a wear time below 60% of the entire 120±15 days (n = 1) or an incomplete recording for any of the five 7-day analysis periods (n = 3) were excluded from analysis. Individual days within a participant’s recording were excluded from analysis if there were fewer than 10 hours of wear during daytime or more than 1 hour of non-wear in the major sleep period (9 pm to 7 am). Data collected April 2020 and later were excluded because of the anticipated effects of the COVID-19 pandemic.

### Neuropsychological testing

Cognitive function was assessed at baseline and at the three-month visit. One participant did not attend both test sessions and was excluded from the analysis, leaving a total of 14 participants (active, n = 8; control, n = 6).

The baseline neuropsychiatric and neuropsychological assessment battery included the Mini Mental State Examination (MMSE; [32]), Montreal Cognitive Assessment (MoCA; [44]), and the following measures from the National Alzheimer’s Coordination Center’s Uniform Data Set version 3.0 [45]: Trail Making Test Part A (TMT-A); TMT Part B; Craft 21 Story Recall: Immediate and Delayed Recall; Number Span Test Forwards; Number Span Test Backwards; Geriatric Depression Scale (GDS); Functional Assessment Scale; Neuropsychiatric Inventory Questionnaire (NPI-Q); and Clinical Dementia Rating (CDR). The Alzheimer’s Disease Assessment Scale-cognitive subscale (ADAS-Cog) was also administered, and sub-scores were calculated for word list immediate and delayed recall [46]. A briefer assessment battery consisting of the MMSE, MoCA, ADAS-Cog, Number Span Test, TMT (A and B), CDR, NPI-Q, and GDS was completed at Month 3 (median of 101 days, range: 85–109).

### Sequencing for APOE status

Genomic DNA was purified from approximately 5x10^6^ peripheral white blood cells using a genomic DNA extraction kit (Qiagen, Hilden, Germany). Extracted DNA was submitted to Genewiz (Cambridge, MA) for SNP genotyping at rs429358, to determine APOE4 allele status. Fourteen participants were genotyped; one participant was excluded from analysis due to an insufficient blood draw.

### Statistical analysis

For EEG analysis, group-level PSD and coherence results were expressed as the median across participants within each group. 95% confidence intervals for the median were obtained by bootstrapping across the participants 60,000 times with replacement. For comparisons between stimulation conditions or participant groups, if a condition or group was involved in more than one family-wise comparison, the Friedman test (for paired comparisons) or the Kruskal-Wallis test (for unpaired comparisons), followed by Dunn’s multiple comparison test was used to calculate p values. Otherwise, the Wilcoxon’s sign rank test (for paired comparisons) or the Mann-Whitney test (for unpaired comparisons) was used. For comparisons at multiple electrode sites or electrodes, the Bonferroni correction was applied by multiplying the p values by the number of electrode sites or electrodes. P values for categorical variables were calculated using Fisher’s exact test.

For MRI analyses, unpaired t-tests were used for group comparisons of ventricular volume while paired t-tests were used for group level analyses of hippocampal volume and mean functional connectivity to seed regions using resting-state fMRI. Functional connectivity data was family-wise error (FWE)-corrected. The voxel-wise p-threshold is p<0.001.

For actigraphy, we performed a 2-way ANOVA (Treatment Group x Time) with Šídák’s multiple comparisons to assess between or within group differences over time. Delta values are the value at a given timepoint minus the baseline value for that variable.

A two-sided p value less than 0.05 was considered significant. Specific statistical tests and parameters are detailed in the figure legends. All statistical analyses were performed using MATLAB 2019b (MathWorks, Natick, MA) or GraphPad Prism 8.4 (GraphPad Software, San Diego, CA).

## Results

### Phase 1 study: Safety and feasibility of using acute (one-time) GENUS for 40Hz entrainment

#### Outpatients recruited at MIT

40Hz light and sound GENUS was safe and well tolerated by all participants for whom adverse effects were available (n = 41), as assessed by an adverse effects questionnaire and by EEG analysis during acute stimulation with the GENUS device (Table 3).

**Table 3:** Adverse events of acute and chronic GENUS stimulation.

Study	Phase 1 study				Phase 2A study		
Adverse events (n, %)	Young CN (n = 13)	Older CN (n = 10)^a^	Mild AD (n = 16)	Epilepsy Patients (n = 2)	Control (n = 7)	Active (n = 8)	p-value
**New Onset Headache**	0	1 (10)	0	0	0	0	
**Dry Eye**	1 (7.6)	0	0	0	0	2 (25)	0.18
**Light Sensitivity**	1 (7.6)	1 (10)	1 (6.2)	0	0	1 (12.5)	0.37
**Nervousness or anxiety**	0	0	0	0	3 (42.8)	1 (12.5)	0.21
**Sleepy or Drowsy**	5 (38.4)	4 (40)	1 (6.2)	0	1 (14.2)	3 (37.5)	0.35
**Numb/ Unfocused**	0	0	0	0	2 (28.5)	2 (25)	0.89
**Bored**	0	0	0	0	0	1 (12.5)	0.37
**Nausea**	1 (7.6)	0	0	0			
**Seizures**	0	0	0	0	0	0	
**≥1 Adverse Event**	1 (7.6)	1 (10)	0	0	2 (28.5)	4 (50)	0.43
**No Adverse Events**	6 (46.1)	5 (50)	14 (87.5)	0	3 (42.8)	3 (37.5)	0.85

Percentages are rounded to the nearest integer. Abbreviations: AD, Alzheimer’s Disease; CN, cognitively normal.

^a^ Two participants not included due to missing data.

**Fig 3:**
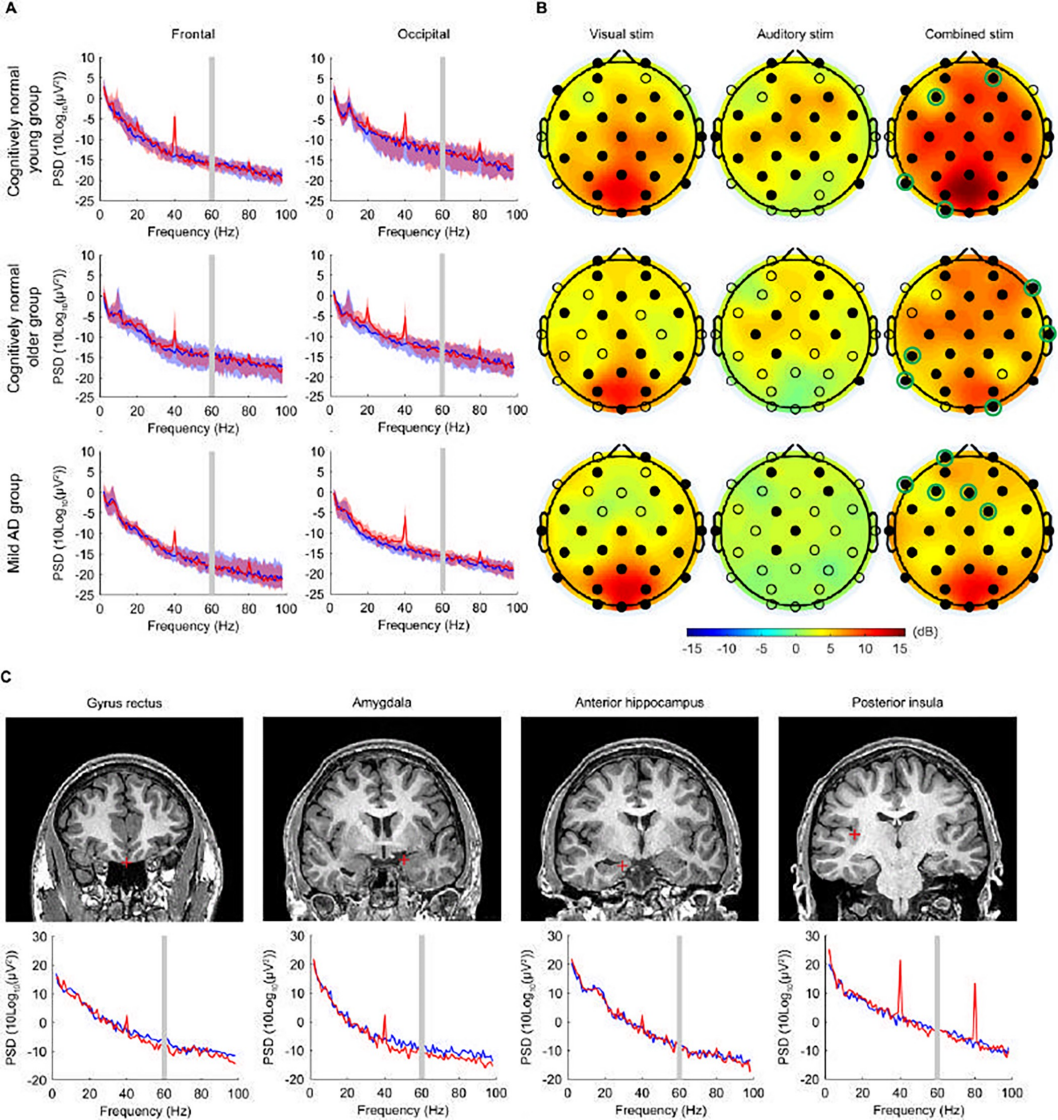
Acute 40Hz combined visual and auditory stimulation entrains cortical and subcortical regions. (A) Scalp EEG power spectral density (PSD) at the frontal (Fz, F3, F4, F7, F8) and the occipital (Oz, O1, O2) electrode sites, in cognitively normal young participants (n = 13; top row), cognitively normal older participants (n = 12; middle row), and patients with mild AD (n = 16; bottom row). Solid lines, group median; shaded areas, 95% confidence interval; blue, baseline; red, 40Hz GENUS light and sound stimulation (active condition). Gray bar indicates 60 Hz line noise. (B) Topographic maps showing the median change in 40Hz PSD from the baseline level with 40Hz visual alone, 40Hz auditory alone, and 40Hz combined stimulation, in cognitively normal young participants (n = 13; top row), cognitively normal older participants (n = 12; middle row), and patients with mild AD (n = 16; bottom row). Filled circles represent scalp electrodes at which the increase in 40Hz PSD from the baseline level was significant (p < 0.05, Wilcoxon’s sign rank test, Bonferroni corrected for 32 electrodes). Green circles indicate electrodes entrained with synchronized 40Hz GENUS light and sound stimulation but not by light or sound alone. (C) Example coronal MRI images before electrode implantation (top row) and intracranial EEG PSD (bottom row) from a single patient with medically intractable epilepsy (Patient 483), for depth electrode contacts placed in the gyrus rectus, amygdala, anterior hippocampus, and posterior insula. Red plus sign, approximate location of the depth electrode contact; blue, baseline; red, 40Hz GENUS light and sound stimulation (active condition). For the PSD between 58Hz and 62Hz, interpolated values are plotted because of the bandstop filtering around 60Hz.

In all groups, 40Hz GENUS light and sound significantly increased the 40Hz PSD relative to the baseline condition at both the frontal and occipital electrode sites (Fig 3A, red line for 40Hz GENUS light and sound vs blue line for baseline), engaging brain regions beyond unimodal sensory regions (Fig 3B, right column). In the mild AD group, electrodes responding specifically to 40Hz GENUS light and sound but not to 40Hz GENUS light or 40Hz GENUS sound (Fig 3B, right column, electrodes with green circles) were grouped in the frontal region (Fp1, F7, F3, Fz, Fc2); in both the young and older cognitively normal groups, these electrodes were spread throughout the brain (young: AF4, F3, P7, O1; older: F8, T8, CP5, P7, O2).

The median change in the 40Hz PSD at the frontal electrode site was 7.71dB (range, 0.68 to 16.88; p < 0.001) for the cognitively normal young group, 7.22dB (range, 0.79 to 13.10; p = 0.002) for the cognitively normal older group, and 5.82dB (range, -0.02 to 10.51; p < 0.001) for the mild AD group; the median change in the 40Hz PSD at the occipital site was 7.74dB (range, 3.51 to 19.45; p < 0.001) for the cognitively normal young group, 7.95dB (range, 1.18 to 14.70; p = 0.002) for the cognitively normal older group, and 7.68dB (range, 4.23 to 18.26; p < 0.001) for the mild AD group. Harmonics at 80Hz and sub-harmonics at 20Hz can be seen in the cognitively normal younger and older groups, and less so in the mild AD group, consistent with previous literature [47]. The increases in 40Hz PSD were accompanied by significant increases in 40Hz coherence between electrode sites (S2A and S2B Fig and S2 Table). Therefore, the combined stimulation induced 40Hz neural oscillations across multiple electrode sites in all groups.

#### Patients with epilepsy recruited at University of Iowa

In the two patients with epilepsy, GENUS was well tolerated with no significant adverse events as evaluated by adverse events questionnaire and by EEG analysis performed by an independent epileptologist (EJS, Table 3). Intracranial EEG showed that 40Hz light and sound GENUS entrains deeper regions of the brain, including the gyrus rectus, amygdala, hippocampus, and insula (Fig 3C) and more superficial areas such as frontal and temporal gyri (S3A and S3B Fig). The largest increases in 40Hz PSD were detected in the insula and the superior temporal cortex, which have been shown to be involved in multi-sensory integration [48, 49]. Across subcortical and deep cortical regions, there was an overall increase in 40Hz PSD during the combined stimulation compared to the baseline period (median [range] change in the 40Hz PSD from the baseline level: 4.58dB [-1.22 to 23.27] in Patient 483; 0.77dB [-1.95 to 6.44] in Patient 493), with the increase in 40Hz PSD appearing at the majority of the electrode contacts (S3A Fig; 75/78 contacts in Patient 483; 10/15 contacts in Patient 493). Additionally, the combined stimulation increased global coherence at 40Hz within deep electrode contacts (S3C Fig).

### Phase 2A study: Safety, feasibility, and exploratory outcomes of using chronic daily GENUS for 40 Hz entrainment

Eightyseven potential participants were screened, of whom 56 did not meet study criteria (S1 Table) due to moderate to severe dementia at screening, 12 declined to participate or did not respond, and 4 did not participate for other reasons. Fifteen participants were enrolled in the longitudinal study, of whom 8 completed the active arm and 7 completed the control arm (CONSORT study flow, Fig 1.). Enrollment is complete for this trial and all remaining participants are now in an open-label long-term extension. Patient characteristics at baseline were not statistically different across the two trial groups, except for years of education, in which the control group had significantly fewer years of education compared to the active group (S4 Fig, Table 2; p = 0.01). However, education was not significantly correlated with any outcome variables of interest (S5 and S6 Figs).

Before being unblinded to condition, participants were asked which intervention they thought they received. In the control group, 33.4% were correct, and in the active group, 50% participants were correct. All patients were offered the opportunity to continue with active stimulation; 12 chose to do so.

#### Safety and compliance of usage

Safety was assessed with EEG during stimulation with the GENUS device at baseline and after 3 months, to monitor for epileptiform discharges, and through weekly phone calls with participants. 40Hz GENUS was well-tolerated by all participants with no significant adverse effects (Table 3). Compliance was measured using timestamp recordings built into the device to indicate when the device was on and with photographic records of participants as they were using the device. After 4 months of daily stimulation, there was no significant difference in compliance between groups–mean usage was 91% ± 7% and 87% ± 9% for the control and active groups, respectively (p = 0.355). Both the control and active groups used their devices with equal compliance at home for an hour daily.

#### Structural MRI

**Fig 4:**
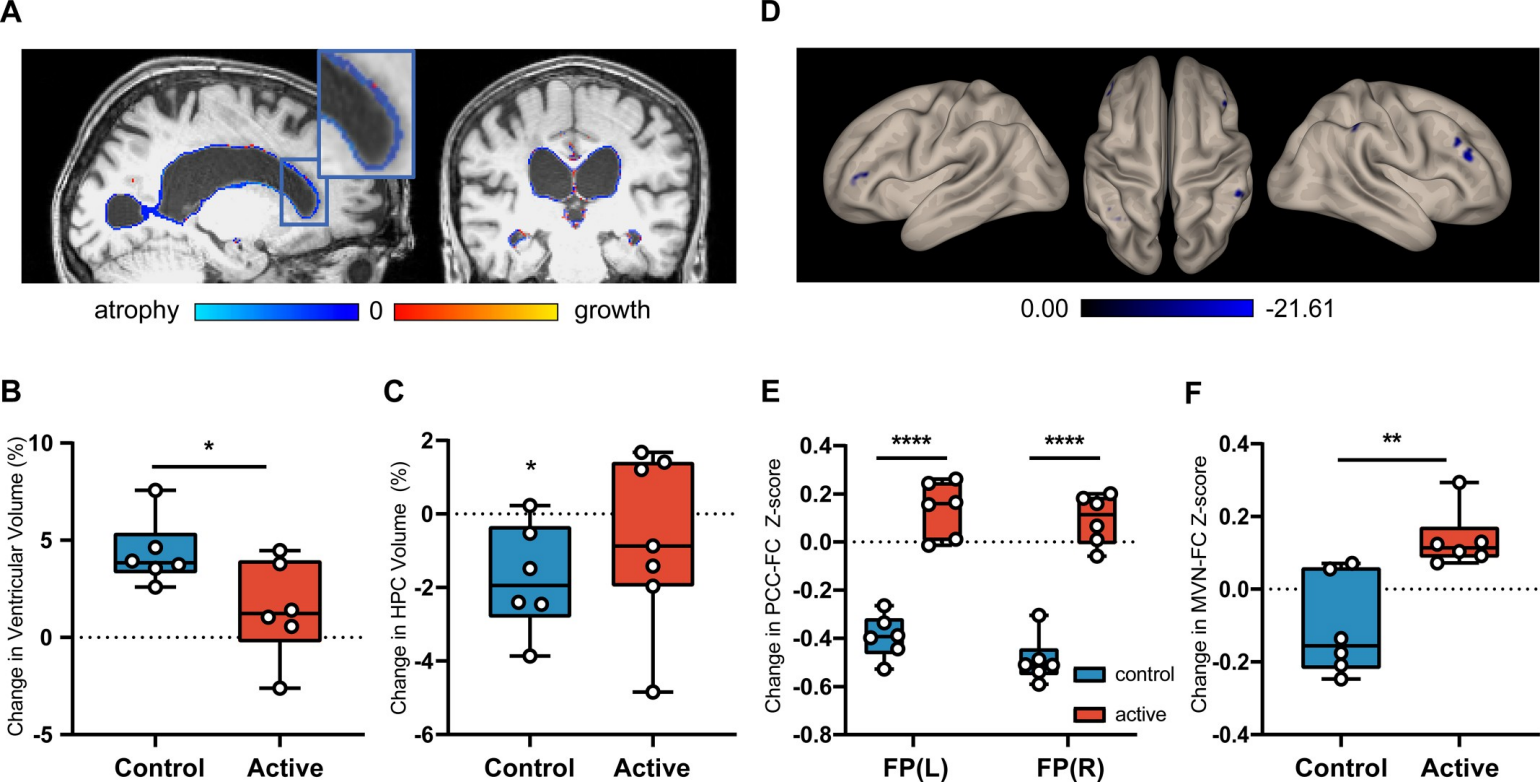
Daily GENUS leads to group-level differences in structural and functional MRI outcomes at Month 3. (A) Visualization of change in ventricular volume of an example control participant from baseline to Month 3 on sagittal and coronal T1-weighted structural MRI (B) Group-level analysis of percent change in ventricular volume between the control and active groups (n = 13, p = 0.024) (C) Group-level analysis of control vs active groups HPC volume compared to 0 (n = 13, control p = 0.034, active p = 0.4384) (D) Seed-to-Voxel analysis of PCC-FC in control group from baseline to Month 3 on resting state fMRI (See S3 Table) (E) Seed-to-voxel analysis of PCC-FC for between group comparisons from baseline to Month 3 (n = 12, p < 0.05 FEW-corrected) (F) Group-level analysis of changes in mean functional connectivity of the MVN from baseline to Month 3. (n = 12, p = 0.004). FP: Frontal Pole; MVN: Medial Visual Network; PCC-FC: Posterior Cingulate Cortex functional connectivity.

Since the natural progression of AD involves cerebral atrophy and accompanying ventricular enlargement, we used structural MRI to test for an effect of GENUS light and sound on ventricular, hippocampal, and total brain volumes. A significant difference in ventricular enlargement between the control (n = 6) and active (n = 7) group at Month 3 was observed (p = 0.024) (Fig 4A and 4B; S5 Table). The control group (n = 6) exhibited ventricular enlargement (4.34 ± 1.72%, p = 0.0016), while the active group (n = 7) had no significant change in ventricular volume (1.33 ± 2.33%) from baseline to Month 3 (p = 0.18); Cohen’s d effect size for the group difference in ventricular enlargement was 0.59. Hippocampal (HPC) volume declined in the control group (-1.75 ± 1.48%, p = 0.034) but not in the active group (-0.69 ± 2.35%, p = 0.438; Fig 4C and S5 Table). Cohen’s d effect size for the group difference in HPC atrophy was 0.26. No significant changes were seen in either group in total brain volume or cortical thickness (S5 Table).

### Functional MRI

To evaluate whether neural networks were altered by 40Hz GENUS light and sound, we used resting-state functional MRI to probe circuits important for memory and sensory processing, including the default mode network (DMN) and medial visual network (MVN), respectively. Participants were removed from analysis if the number of valid scans made up less than 10% of the total recorded scans due to motion or other artifacts (n = 1 removed, see CONSORT diagram Fig 1). A seed-to-voxel analysis of the posterior hub of the DMN containing the posterior cingulate cortex (PCC) and the precuneus was performed and differences between baseline and follow-up scans were compared for each individual group, as well as between groups. The control group (n = 6) had declines in functional connectivity between the posterior hub of the DMN and the left frontal pole, posterior right supramarginal gyrus, left angular gyrus, inferior right frontal gyrus, and superior right frontal gyrus (p<0.05 FWE-corrected, voxel-wise p-threshold is p<0.001; Fig 4D, S3 Table). The active group (n = 6) did not show any significant change in connectivity with the PCC after 3 months of GENUS. Analysis of the connectivity between the PCC and the bilateral frontal poles showed a significant difference between groups in the change from baseline to Month 3 due to reduced connectivity between these regions in the control group (p = 0.031 and p = 0.032 for left and right respectively, FWE-corrected, peak T = 7.32 and 7.01, respectively; Fig 4E, S3 Table).

The active group showed a significant increase in mean functional connectivity of the MVN with the rest of the brain at Month 3 (Fig 4F; 0.14 ± 0.07, p = 0.009); the control group did not show this change (-0.11 ± 0.14). No difference was observed in baseline values (S4 Table). When analyzed with the hippocampus as the seed region, functional connectivity in the active group increased significantly between the left hippocampus and the visual cortex (p = 0.001 and p = 0.006 for left and right lateral occipital cortex (superior), respectively, FWE-corrected, peak T = 12.64 and 17.37, respectively) while the control group did not show a change (S3 Table).

Task-based fMRI data did not show any differences between the control and active groups during the visual presentation of novel face-name vs repeated face-name pairs (see source data).

#### Daily rhythmicity in activity

Prospective studies have demonstrated reduced stability and increased fragmentation of daily rhythms with age, an increased risk of such rhythm disturbances with diagnosis of mild cognitive impairment (MCI) or AD, and an accelerated progression from MCI to AD in the presence of such rhythm disturbances [50]. We therefore assessed how GENUS impacts daily rhythms, using objective actigraphy as a measure. We derived two standardized non-parametric measures from the actigraphy recordings, inter-daily stability (IS) and intra-daily variability (IV). IS is defined as the ratio between the variance of the average 24-h pattern and the overall variance and measures day-to-day consistency of activity rhythms (e.g., bedtime schedule or ability to become active at a regular time); this measure is a sign of robust coupling to environmental cues. IV is defined as the ratio between the mean square first derivative and the overall variance [51] and measures the robustness of the daily rhythms. IS and IV are fixed values ranging between 0–1 and 0–2, respectively.

**Fig 5:**
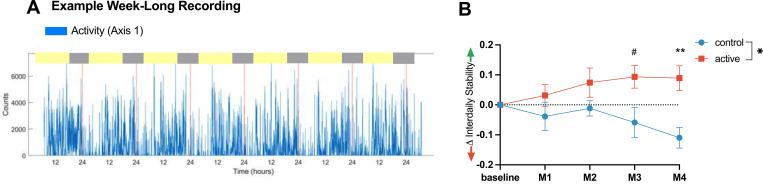
Changes in rest-activity patterns relative to baseline. (A) Example 7-day activity recording of activity counts (blue) with red dotted lines every 24 hours from an individual. Inter-daily Stability (IS) quantifies the regularity of day-to-day rest-activity patterns. (B) Improved IS compared to baseline (means ± SEM) in active group (red) but not control group (blue), with significant differences between active and control groups at Month 4 (**p < 0.01) and a trend at Month 3 (#p < 0.1). Statistical analysis with a 2-Way ANOVA with Šídák’s multiple comparisons at each monthly timepoint. Mean of control = -0.055, mean of active = 0.072. Main effect of intervention p = 0.037, interaction of intervention x time, p = 0.025. Multiple comparison at Month 3, p = 0.051, Month 4, p = 0.007.

At baseline, there were no differences between active and control groups in IS (active mean 0.52, control mean 0.63, p = 0.83) or IV (active mean 1.13, control mean 0.86, p = 0.4). The active group had improved IS over the first 4 months and significantly improved IS compared to the control group after 4 months (active mean 0.072, SD = 0.028; control mean -0.055, SD = 0.041; p = 0.036, interaction p = 0.025 with Šídák’s multiple comparisons at M3 p = 0.051 and M4 p = 0.007) (Fig 5). The IV of both groups did not significantly change from baseline (S7 Fig).

### Cognition

Cognitive function was assessed at baseline and again at the Month 3 visit. There were no significant differences between groups in cognitive functioning as assessed using the MMSE (p = 0.536), MoCA (p = 0.198), ADAS-Cog (p = 0.237), or CDR Rating (p = 0.792); this was expected given the short (i.e., 3 months) study time frame (Table 4).

**Table 4:** Changes in clinical characteristics in Phase 2A study participants after 3 months.

Group	Timepoint	MMSE^a^	MoCA^a^	ADAS-Cog^b^	FNA-DRT	Global CDR^c^	
						1.0	0.5
	median (range)					n (%)	
Control (n = 7)	Baseline	22.0 (18.0–24.0)	15.0 (7.0–20.0)	19.00 (10.33–24.00)	8.0^e^ (6–10)	6 (86)	1 (14)
Control (n = 6)^d^	Month 3	21.0 (17.0–24.)	14.05 (12.0–17.0)	19.33 (8.00–30.66)	6.0^e^ (4–12)	2 (33)	4 (67)
**Intra-group change (p-value)**		0.72	0.69	0.78	0.53	0.05	
Active (n = 8)	Baseline	23.5 (19.0–27.0)	19.5 (14.0–23.0)	15.00 (6.66–30.00)	5.5^f^ (5–9)	5 (63)	3 (37)
Active (n = 8)	Month 3	22.5 (19.0–30.0)	20.0 (15.0–29.0)	15.50 (3.33–20.66)	9.5^f^ (9–12)	4 (50)	4 (50)
**Intra-group change (p-value)**		0.56	0.05	0.32	0.004**	0.61	
**Inter-group change (p-value)**	Baseline	0.32	0.09	0.26	0.23	0.31	
	Month 3	0.54	0.2	0.24	0.027*	0.79	

Percentages are rounded to the nearest integer. Abbreviations: ADAS-Cog, Alzheimer’s Disease Assessment Scale-Cognitive Subscale; CDR, Clinical Dementia Rating; FNA-DRT, Face-Name Association Delayed Recall Test; MMSE, Mini Mental State Examination; MoCA, Montreal Cognitive Assessment.

^a^ Ranges from 0 to 30, with a higher score indicating less impairment.

^b^ Ranges from 0 to 70, with a higher score indicating greater impairment.

^c^ Ranges from 0 to 3, where 0 = normal, 0.5 = very mild dementia, 1 = mild dementia, 2 = moderate dementia, 3 = severe dementia

^d^ Only n = 6 control participants completed Month 3 cognitive assessments due to COVID-19 shut-down

^e^ n = 5

^f^ n = 4

**Fig 6:**
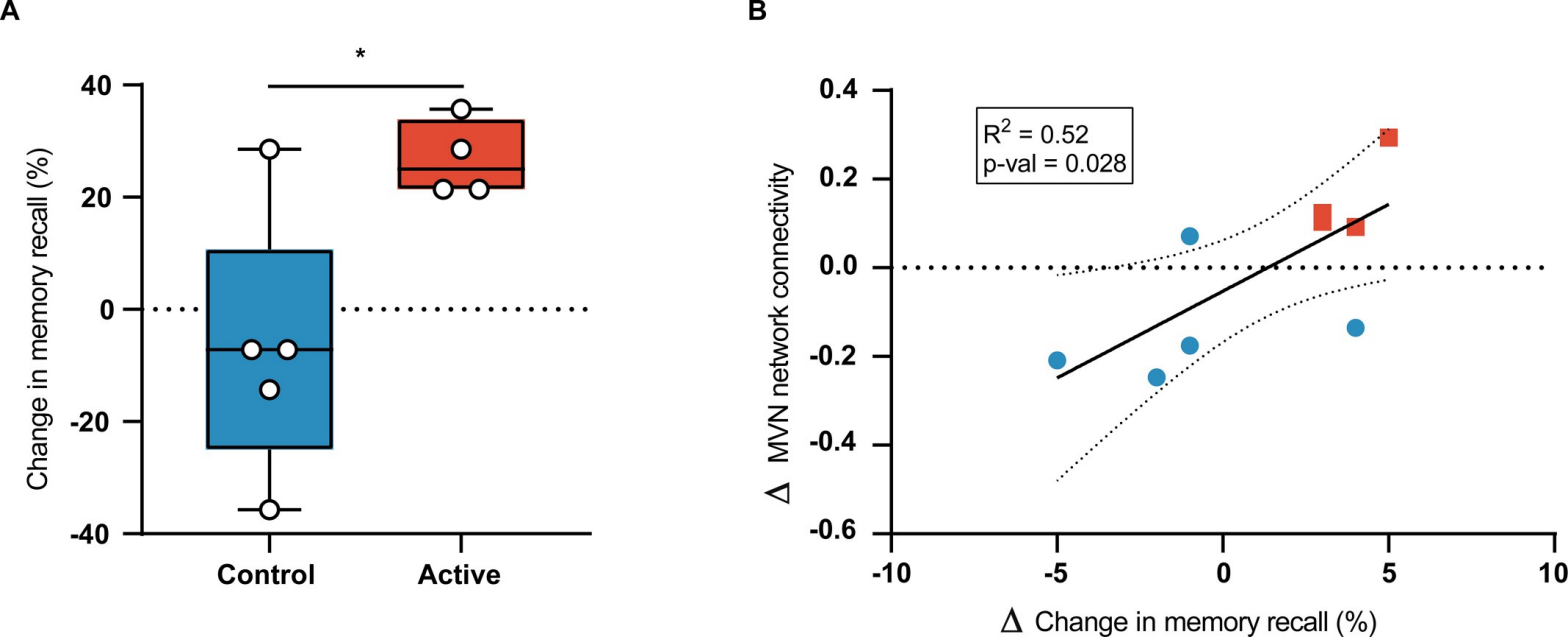
Chronic GENUS improves face-name association delayed recall test (FNA-DRT) results. (A) Group-level analysis of changes in FNA-DRT from baseline to Month 3 (B) Correlation of change in FNA-DRT score with change in mean functional connectivity of the MVN.

At the Month 3 visit, the active group (n = 4) had a significant improvement in accuracy on the FNA-DRT (Fig 6A; 3.75 ± 0.96 pts, p = 0.004), while no significant change was observed in the control group (n = 5) (Fig 6A; -1 ± 3.24 pts, p = 0.528); this difference in accuracy in the FNA-DRT between groups was statistically significant (p = 0.027). When data from both groups were pooled, we found that improvement in accuracy on the FNA-DRT was correlated with increased functional connectivity of the MVN (Fig 6B; R^2^ = 0.52, R = 0.772, p = 0.028).

### Post-hoc testing for effect of years of education

Years of education did not correlate with any outcome measures reported, including the FNA-DRT (R^2^ 0.0003, p = 0.96), connectivity with MVN (R^2^ = 0.097, p = 0.33), change in hippocampal volume (R^2^ = 0.16, p = 0.18), change in connectivity with PCC (R^2^ = 0.007, p = 0.79), or change in ventricular volume (R^2^ = 0.19, p = 0.15) (S5 Fig).

## Discussion

Our Phase 1 study demonstrated that 40Hz GENUS using synchronized light and sound can effectively induce gamma entrainment across multiple brain regions in cognitively normal individuals, patients with medically intractable epilepsy, and in patients with mild AD dementia. We are the first to show that GENUS light and sound stimulation entrains not only cortical sensory regions, but also distant cortical and subcortical regions such as the gyrus rectus, the amygdala, the hippocampus, and, in particular, the insula. Scalp electrodes showing entrainment with the combined GENUS light and sound stimulation were distributed differently in different participant groups: across multiple areas in the cognitively normal young and older groups but concentrated around frontal regions in the mild AD dementia group. This difference among the groups may reflect age- or disease-related changes in neural responses to sensory stimulation, which could potentially be leveraged as a biomarker for aging or AD [52, 53]. Finally, our Phase 1 study showed that 40Hz GENUS did not trigger epileptiform activity even in patients with epilepsy and did not cause any severe adverse effects.

Our Phase 2A study demonstrated that compliance of usage was equal between the control and active groups. Neither group accurately guessed their GENUS settings prior to unblinding (control group guessed correctly 33.4%, active group 50%), which supports our ability to blind participants effectively to study condition. We found that, relative to the control group, the active group that had daily usage of 40Hz GENUS over 3 months had less brain atrophy and reduced loss of functional connectivity, improved markers of sleep, and improved performance on an associative memory task. Overall, these findings suggest that 40Hz GENUS has positive effects on AD-related pathology and symptoms and should be studied more extensively to evaluate its potential as a disease-modifying intervention for AD.

AD is associated with ventricular expansion and hippocampal atrophy. The degree of ventricular expansion and hippocampal atrophy experienced by the control group in our study is consistent with previous studies of mild AD dementia (56, 57). A previous study observing the rate of ventricular expansion over a period of 6 months found that participants with AD saw an increase of 5.7±4.9% over the observation period [55]. The rate of change observed in our control group (4.34±1.72%) falls within the reported range. In contrast, no significant change in ventricular or hippocampal volume was seen in the active group receiving combined GENUS light and sound stimulation, in agreement with findings in AD animal models [20]. Given that ventricular expansion and hippocampal atrophy correlate with cognitive function and clinical disease progression, this suggests the possibility that neurodegeneration may have slowed in the active group [54–56].

Resting-state fMRI has been used to examine functional brain network disruptions in patients with AD that correlate with cognition [57, 58]. The network that shows the greatest amount of dysfunction in AD is the DMN, which exhibits hypoconnectivity in patients with AD, MCI, and even those at high risk for AD, especially within its major hub–the PCC [59–61]. This region plays a critical role in attention and internally-directed thought and is connected with the medial temporal lobe system and correlated with memory encoding and recall [62–64]. Consistent with changes seen in the DMN in the natural progression of AD [65–67], our control group had significant loss of connectivity between the PCC and several regions in the frontal cortex, as well as the angular gyrus. In contrast, the active group receiving combined GENUS light and sound stimulation did not show connectivity changes within the DMN.

Given the visual component in our intervention, we also quantified the functional connectivity of the visual network, even though this is not typically evaluated in resting-state fMRI studies in AD research. We found a significant increase in mean functional connectivity in the MVN in the active group but not in the control group. Our work in animal models showed that GENUS impacted oscillatory activity and amyloid deposition within the sensory cortices related to a given stimulus and that visual stimuli resulted in a significantly greater EEG response than auditory stimuli [17, 21]. Therefore, 3 months of daily 40Hz GENUS light and sound stimulation may specifically impact AD pathology in brain regions processing visual stimuli, leading to the observed increase in mean functional connectivity in the MVN. It is less likely that increased MVN connectivity simply resulted from daily use of the light panel independent of the 40Hz stimulation, because we did not see the increase in our control group.

Not surprisingly, performance on cognitive tasks such as the MOCA, MMSE, ADAS-Cog did not show significant changes over the short 3-month period of daily intervention. Improvement was only seen in the FNA-DRT, which has been reported to be a sensitive marker of early amyloid-related memory impairment (42). Associative memory tasks in general have been found to be especially sensitive in the early stages of AD [68–71]. Another group using TACS at gamma frequency in a double-blind, sham controlled crossover pilot study in 20 participants with MCI due to AD showed that a single session of stimulation resulted in significant improvements in FNA-DRT scores and episodic memory [72]. Given that we observed a significant increase in the connectivity between the hippocampus and the visual cortex in the active group but not in the control group, it is also intriguing to speculate that GENUS light stimulation may selectively improve visually evoked memory.

Prospective studies of daily activity rhythms demonstrate reduced stability and increased fragmentation with age and show a bi-directional relationship between these rhythm disturbances and AD. Disturbances in daily activity rhythms accelerate the progression from MCI to AD, and a diagnosis of MCI or AD is associated with accelerated worsening of the disturbances [50]. Our findings of significantly increased day-to-day regularity of activity patterns (IS) over four months in the active group but not the control group are consistent with findings that GENUS light and sound can intervene to improve daily rhythms that ordinarily degrade with age and in AD [31, 73]. Sleep may also be affected by combined GENUS stimulation. Future GENUS work should include formal studies of sleep using polysomnography and explore any relationship between sleep metrics (e.g., timing, duration) and clearance of AD-related pathology such as cerebral amyloid and tau.

Our studies are the first to show that 40Hz GENUS induced gamma entrainment can be seen not only in cortical brain regions but also in distant cortical and in subcortical areas including the hippocampus and insula and that it is safe and tolerable for patients with mild AD dementia. We report preliminary convergent evidence that 40Hz GENUS may result in positive cognitive and biomarker outcomes, including increased mean connectivity to nodes in the DMN. The major limitation of our Phase 2A study is the small sample size compounded by some missing data, and we recognize that participants may have exhibited the observed changes without the intervention given the heterogeneity of the rate of progression of AD. In addition, the COVID-19 pandemic interfered with data collection for MRI and EEG after 3 months of stimulation. Nevertheless, our study supports investigation of 40Hz GENUS in larger, longer clinical trials in AD to determine whether the findings reported here are robust and reproducible and to investigate effects of 40Hz GENUS on amyloid and tau biomarkers of AD.

## Supporting information

S1 Checklist CONSORT 2010 checklist of information to include when reporting a pilot or feasibility randomized trial in a journal or conference abstract. (DOC)

S1 Fig Study timelines. (PNG)

S2 Fig Changes in scalp EEG power spectral density and coherence with acute 40Hz combined stimulation, related to Fig 1. (A) Scalp EEG power spectral density (PSD) at the central (Cz, C3, C4) and parietal (Pz, P3, P4, P7, P8) electrode sites, in cognitively normal young participants (n = 13; top row), cognitively normal older participants (n = 12; middle row), and patients with mild AD (n = 16; bottom row). Solid lines, group median; shaded areas, 95% confidence interval; blue, baseline; red, GENUS light and sound. Gray bar placed around frequency range with 60Hz line noise. (B) Scalp EEG global coherence in cognitively normal young participants (n = 13; top row), cognitively normal older participants (n = 12; middle row), and patients with mild AD (n = 16; bottom row). Solid lines, group median; shaded areas, 95% confidence interval; blue, baseline; red, GENUS light and sound. Gray bar placed around frequency range with 60Hz line noise.
(PNG)

S3 Fig Intracranial EEG power spectral density and coherence during baseline and acute 40Hz combined stimulation, related to Fig 1. (A, B) Example coronal MRI images before electrode implantation (top row) and intracranial EEG power spectral density (PSD; bottom row) from the patients with epilepsy (Patient 483 (A) and Patient 493 (B)) for depth electrode contacts placed in deep and superficial brain regions. White plus sign, approximate location of the depth electrode contact; blue, baseline; red, 40Hz combined stimulation. For the PSD between 58Hz and 62Hz, interpolated values are plotted, because of bandstop filtering around 60Hz. (C) Global coherence in Patient 483 (top panel) and Patient 493 (bottom panel). Blue, baseline; red, 40Hz GENUS light and sound. For the global coherence between 58Hz and 62Hz, interpolated values are plotted, because of bandstop filtering around 60Hz. Change in global coherence at 40Hz on baseline recording was 0.26 for Patient 483 and 0.09 for Patient 493.
(PNG)

S4 Fig Participant’s years of education. Results of an unpaired t-test, *p < 0.05. p = 0.012 (n = 15, control = 7, active = 8).
(PNG)

S5 Fig Years of education related to outcomes of the face-name association delayed recall test (FNA-DRT) and MRI. (A) Simple linear regression of participant’s level of education (years) vs change in FNA-DRT. Goodness of Fit R^2^ = 0.0003, p = 0.96. Solid black line represents best fit line, dotted black represents 95% Confidence Interval. (B) Simple linear regression of participant’s level of education (years) vs change in Medial Visual Network Connectivity. Goodness of Fit R^2^ = 0.097, p = 0.33. Solid black line represents best fit line, dotted black represents 95% Confidence Interval. (C) Simple linear regression of participant’s level of education (years) vs change in Bilateral Hippocampal Volume. Goodness of Fit R^2^ = 0.16, p = 0.18. Solid black line represents best fit line, dotted black represents 95% Confidence Interval. (D) Simple linear regression of participant’s level of education (years) vs change in Posterior Cingulate Cortex Connectivity. Goodness of Fit R^2^ = 0.007, p = 0.79. Solid black line represents best fit line, dotted black represents 95% Confidence Interval. (E) Simple linear regression of participant’s level of education (years) vs change in Ventricular Volume. Goodness of Fit R^2^ = 0.19, p = 0.15. Solid black line represents best fit line, dotted black represents 95% Confidence Interval.
(PNG)

S6 Fig Years of education related to cognition at baseline and change in cognition at Month 3 visit. (A) Simple linear regression of participant’s level of education (years) vs baseline Mini-Mental Status Exam (MMSE). Goodness of Fit R^2^ = 0.066, p = 0.35. Solid black line represents best fit line, dotted black represents 95% Confidence Interval. (B) Simple linear regression of participant’s level of education (years) vs baseline Montreal Cognitive Assessment (MoCA). Goodness of Fit R^2^ = 0.0055, p = 0.4. Solid black line represents best fit line, dotted black represents 95% Confidence Interval. (C) Simple linear regression of participant’s level of education (years) vs baseline Alzheimer’s Disease Assessment Scale (ADAS). Goodness of Fit R^2^ = 0.084, p = 0.29. Solid black line represents best fit line, dotted black represents 95% Confidence Interval. (D) Simple linear regression of participant’s level of education (years) vs change in MMSE. Goodness of Fit R^2^ = 6.180e-005, p = 0.98. Solid black line represents best fit line, dotted black represents 95% Confidence Interval. (E) Simple linear regression of participant’s level of education (years) vs change in MoCA. Goodness of Fit R^2^ = 0.096, p = 0.28. Solid black line represents best fit line, dotted black represents 95% Confidence Interval. (F) Simple linear regression of participant’s level of education (years) vs change in ADAS. Goodness of Fit R^2^ = 0.2, p = 0.11. Solid black line represents best fit line, dotted black represents 95% Confidence Interval.
(PNG)

S7 Fig Actigraphy data for circadian rhythm fragmentation. Change in Intradaily Variability demonstrates a trend towards stabilization in the active group but no change in control group. Statistical analysis with a 2-Way ANOVA with Šídák’s multiple comparisons at each timepoint compared to 0 (no-change). Lines indicate means ± SEM. Mean of control = 0.13, mean of active = -0.04. Main effect of intervention, p = 0.22, interaction of intervention x time, p = 0.88, M4 multiple comparison, control, p = 0.30, active, p = 0.99.
(PNG)

S1 Table Eligibility criteria. (PDF)

S2 Table Changes in scalp EEG global coherence at 40Hz with acute 40Hz combined stimulation, related to Fig 1. (PDF)

S3 Table Resting-state functional connectivity clusters. From top to bottom indicating regions showing change in functional connectivity with the posterior hub of the default mode network including the posterior cingulate cortex (PCC) and precuneus, change in control group connectivity between sessions, change in active group connectivity between sessions, and change between groups between sessions, followed by functional connectivity changes for left and bilateral hippocampus. Right hippocampus showed no changes across all comparisons. P < 0.05 FWE-corrected for all clusters, paired T-test. SMG: Supramarginal Gyrus; FG: Frontal Gyrus; AG: Angular Gyrus; LOC: Lateral Occipital Cortex; FP: Frontal Pole; OP: Occipital Pole.
(PDF)

S4 Table Baseline comparisons between groups for MRI related outcomes. (T-test, mean (+/-std)); HPC: Hippocampus; MVN: Medial Visual Network; FC: Functional Connectivity; FNA: Face-name association.
(PDF)

S5 Table Structural MRI statistics. p-values generated using paired T-test.
(PDF)

S1 Protocol (PDF)

S2 Protocol (PDF)

S3 Protocol (PDF)
