# Supplementary material for: Gamma frequency sensory stimulation in mild probable Alzheimer’s dementia patients: Results of feasibility and pilot studies
Source: PLoS One. 2022 Dec 1;17(12):e0278412. doi: 10.1371/journal.pone.0278412 (PMC9714926; doi:10.1371/journal.pone.0278412)
Supplement: S1 Checklist — (DOC) [file pone.0278412.s001.doc]

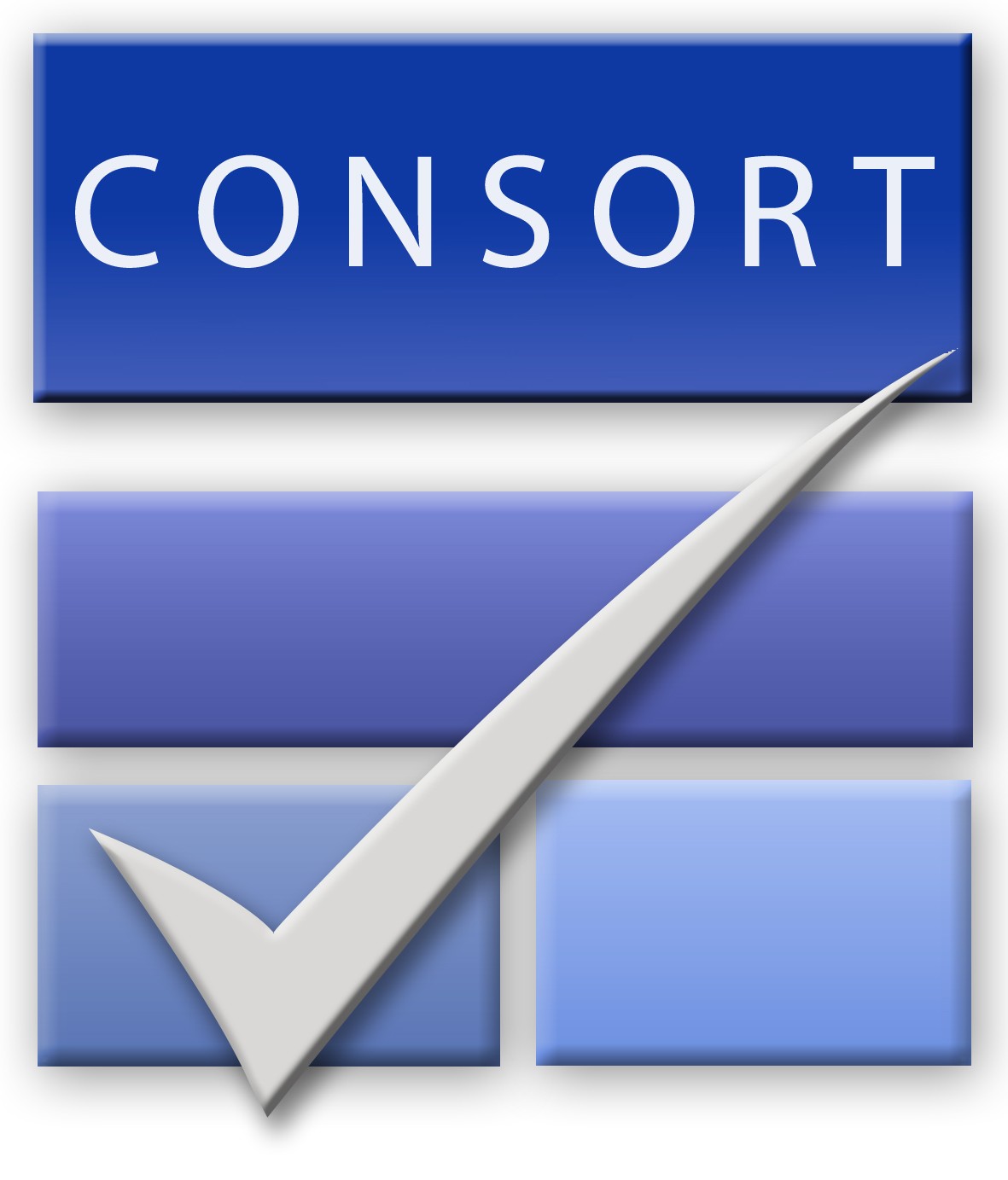
**CONSORT 2010 checklist of information to include when reporting a pilot or feasibility randomized trial in a journal or conference abstract**

| **Item** | **Description** | **Reported on line number** |
| --- | --- | --- |
| Title | Identification of study as randomised pilot or feasibility trial | 1 |
| Authors * | Contact details for the corresponding author | 56 |
| Trial design | Description of pilot trial design (eg, parallel, cluster) | 64 |
| Methods |  |  |
| Participants | Eligibility criteria for participants and the settings where the pilot trial was conducted | 199 |
| Interventions | Interventions intended for each group | 236 |
| Objective | Specific objectives of the pilot trial | 66, 69, 144 |
| Outcome | Prespecified assessment or measurement to address the pilot trial objectives** | 164, 179 |
| Randomization | How participants were allocated to interventions | 168 |
| Blinding (masking) | Whether or not participants, care givers, and those assessing the outcomes were blinded to group assignment | 169, 187 |
| Results |  |  |
| Numbers randomized | Number of participants screened and randomised to each group for the pilot trial objectives** | 480 |
| Recruitment | Trial status† | 484 |
| Numbers analysed | Number of participants analysed in each group for the pilot objectives** | 195, 410, 429, 463, 483 |
| Outcome | Results for the pilot objectives, including any expressions of uncertainty** | 629, 643, 716 |
| Harms | Important adverse events or side effects | 253 |
| Conclusions | General interpretation of the results of pilot trial and their implications for the future definitive trial | 649 |
| Trial registration | Registration number for pilot trial and name of trial register | 153 |
| Funding | Source of funding for pilot trial | All funding sources listed in financial disclosure statement |

Citation: Eldridge SM, Chan CL, Campbell MJ, Bond CM, Hopewell S, Thabane L, et al. CONSORT 2010 statement: extension to randomised pilot and feasibility trials. BMJ. 2016;355.

**this item is specific to conference abstracts*

***Space permitting, list all pilot trial objectives and give the results for each. Otherwise, report those that are a priori agreed as the most important to the decision to proceed with the future*

*definitive RCT.*

*†For conference abstracts.*
