# Supplementary figures and images for: Gamma frequency sensory stimulation in mild probable Alzheimer’s dementia patients: Results of feasibility and pilot studies
Source: PLoS One. 2022 Dec 1;17(12):e0278412. doi: 10.1371/journal.pone.0278412 (PMC9714926; doi:10.1371/journal.pone.0278412)

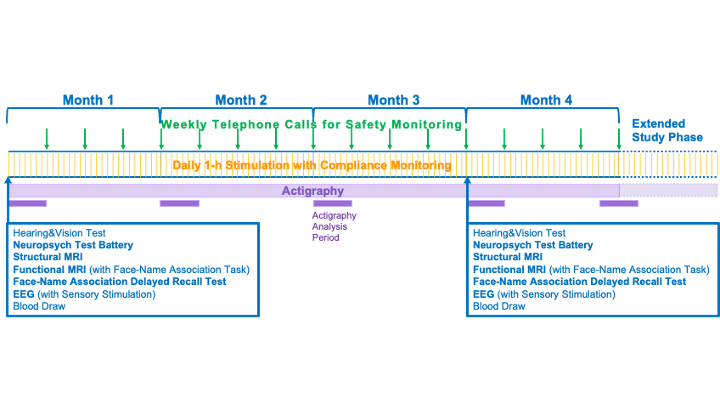

Supplement: S1 Fig — (PNG) [file pone.0278412.s002.png]

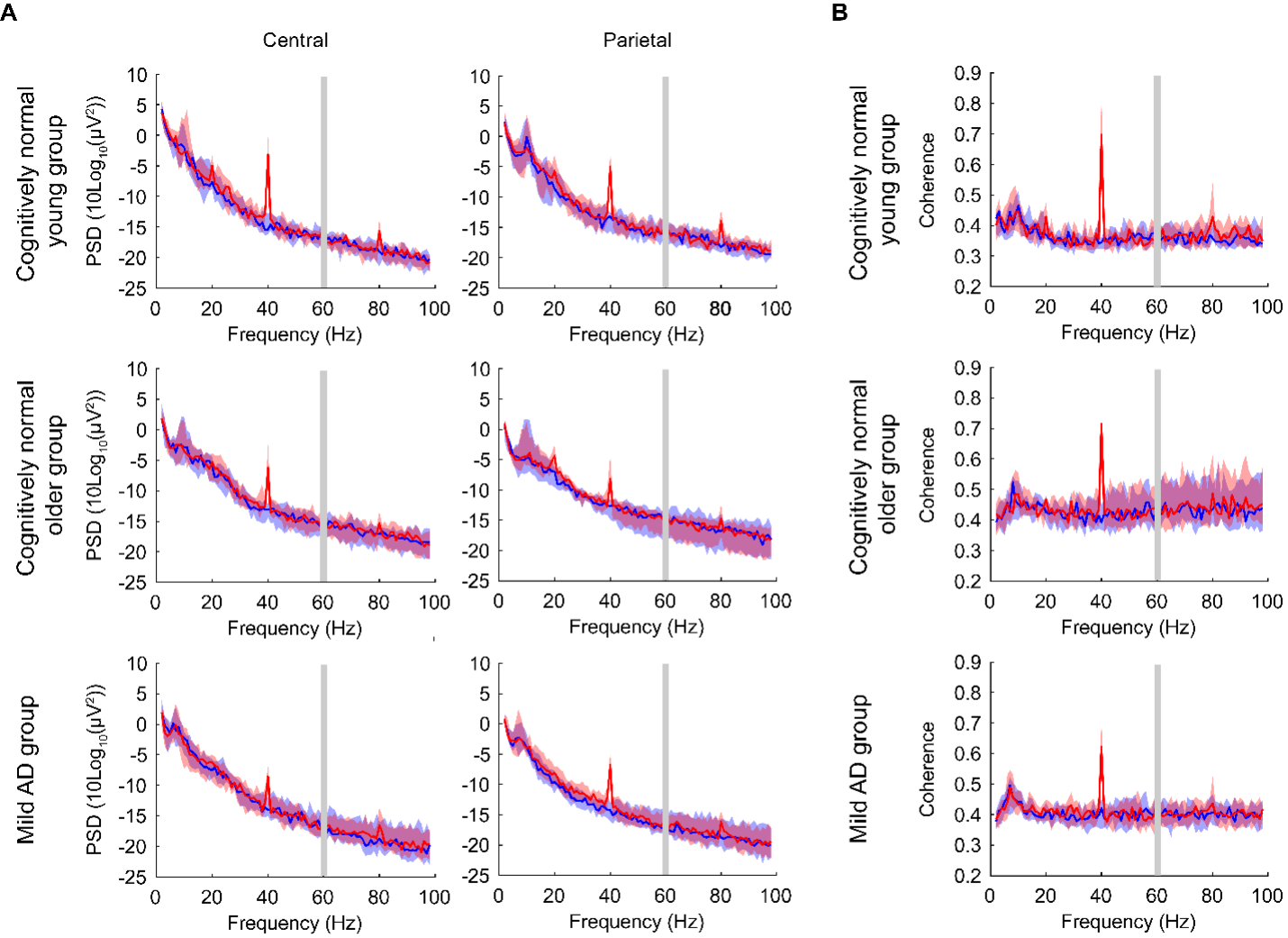

Supplement: S2 Fig — (A) Scalp EEG power spectral density (PSD) at the central (Cz, C3, C4) and parietal (Pz, P3, P4, P7, P8) electrode sites, in cognitively normal young participants (n = 13; top row), cognitively normal older participants (n = 12; middle row), and patients with mild AD (n = 16; bottom row). Solid lines, group median; shaded areas, 95% confidence interval; blue, baseline; red, GENUS light and sound. Gray bar placed around frequency range with 60Hz line noise. (B) Scalp EEG global coherence in cognitively normal young participants (n = 13; top row), cognitively normal older participants (n = 12; middle row), and patients with mild AD (n = 16; bottom row). Solid lines, group median; shaded areas, 95% confidence interval; blue, baseline; red, GENUS light and sound. Gray bar placed around frequency range with 60Hz line noise. (PNG) [file pone.0278412.s003.png]

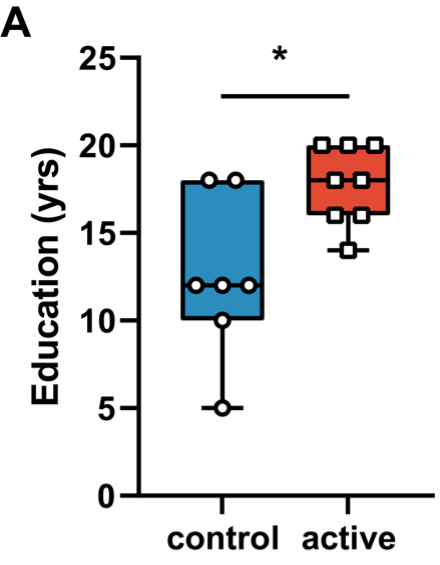

Supplement: S4 Fig — Results of an unpaired t-test, *p < 0.05. p = 0.012 (n = 15, control = 7, active = 8). (PNG) [file pone.0278412.s005.png]

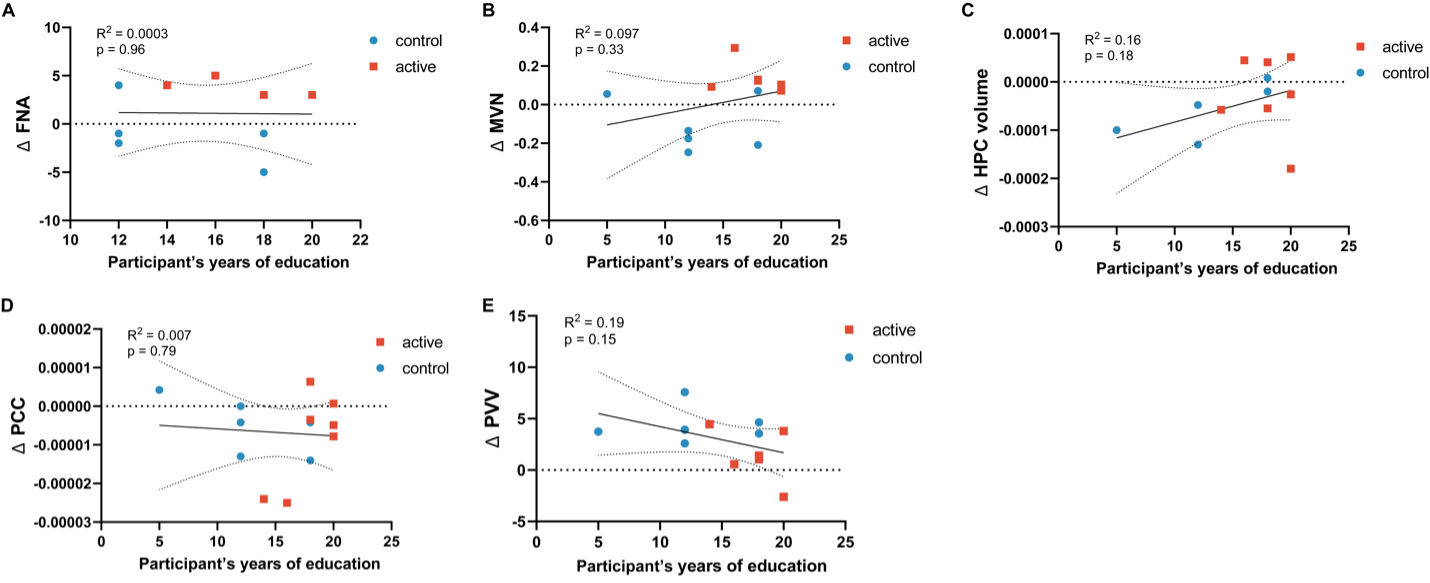

Supplement: S5 Fig — (A) Simple linear regression of participant’s level of education (years) vs change in FNA-DRT. Goodness of Fit R2 = 0.0003, p = 0.96. Solid black line represents best fit line, dotted black represents 95% Confidence Interval. (B) Simple linear regression of participant’s level of education (years) vs change in Medial Visual Network Connectivity. Goodness of Fit R2 = 0.097, p = 0.33. Solid black line represents best fit line, dotted black represents 95% Confidence Interval. (C) Simple linear regression of participant’s level of education (years) vs change in Bilateral Hippocampal Volume. Goodness of Fit R2 = 0.16, p = 0.18. Solid black line represents best fit line, dotted black represents 95% Confidence Interval. (D) Simple linear regression of participant’s level of education (years) vs change in Posterior Cingulate Cortex Connectivity. Goodness of Fit R2 = 0.007, p = 0.79. Solid black line represents best fit line, dotted black represents 95% Confidence Interval. (E) Simple linear regression of participant’s level of education (years) vs change in Ventricular Volume. Goodness of Fit R2 = 0.19, p = 0.15. Solid black line represents best fit line, dotted black represents 95% Confidence Interval. (PNG) [file pone.0278412.s006.png]

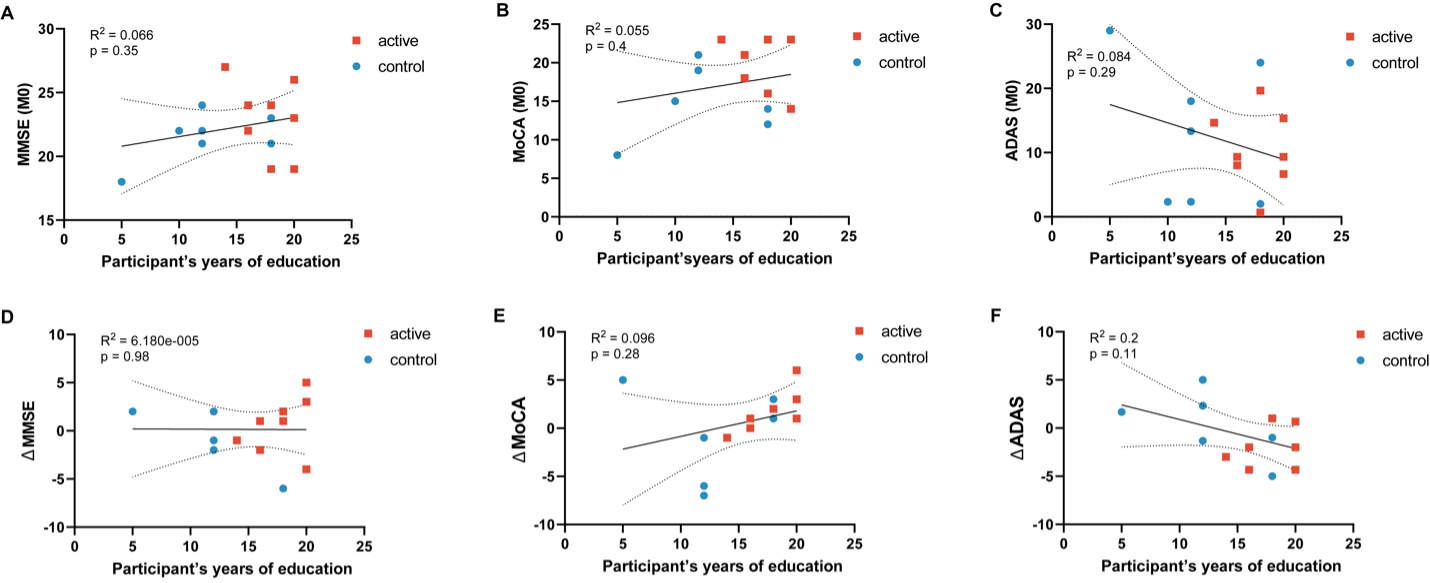

Supplement: S6 Fig — (A) Simple linear regression of participant’s level of education (years) vs baseline Mini-Mental Status Exam (MMSE). Goodness of Fit R2 = 0.066, p = 0.35. Solid black line represents best fit line, dotted black represents 95% Confidence Interval. (B) Simple linear regression of participant’s level of education (years) vs baseline Montreal Cognitive Assessment (MoCA). Goodness of Fit R2 = 0.0055, p = 0.4. Solid black line represents best fit line, dotted black represents 95% Confidence Interval. (C) Simple linear regression of participant’s level of education (years) vs baseline Alzheimer’s Disease Assessment Scale (ADAS). Goodness of Fit R2 = 0.084, p = 0.29. Solid black line represents best fit line, dotted black represents 95% Confidence Interval. (D) Simple linear regression of participant’s level of education (years) vs change in MMSE. Goodness of Fit R2 = 6.180e-005, p = 0.98. Solid black line represents best fit line, dotted black represents 95% Confidence Interval. (E) Simple linear regression of participant’s level of education (years) vs change in MoCA. Goodness of Fit R2 = 0.096, p = 0.28. Solid black line represents best fit line, dotted black represents 95% Confidence Interval. (F) Simple linear regression of participant’s level of education (years) vs change in ADAS. Goodness of Fit R2 = 0.2, p = 0.11. Solid black line represents best fit line, dotted black represents 95% Confidence Interval. (PNG) [file pone.0278412.s007.png]

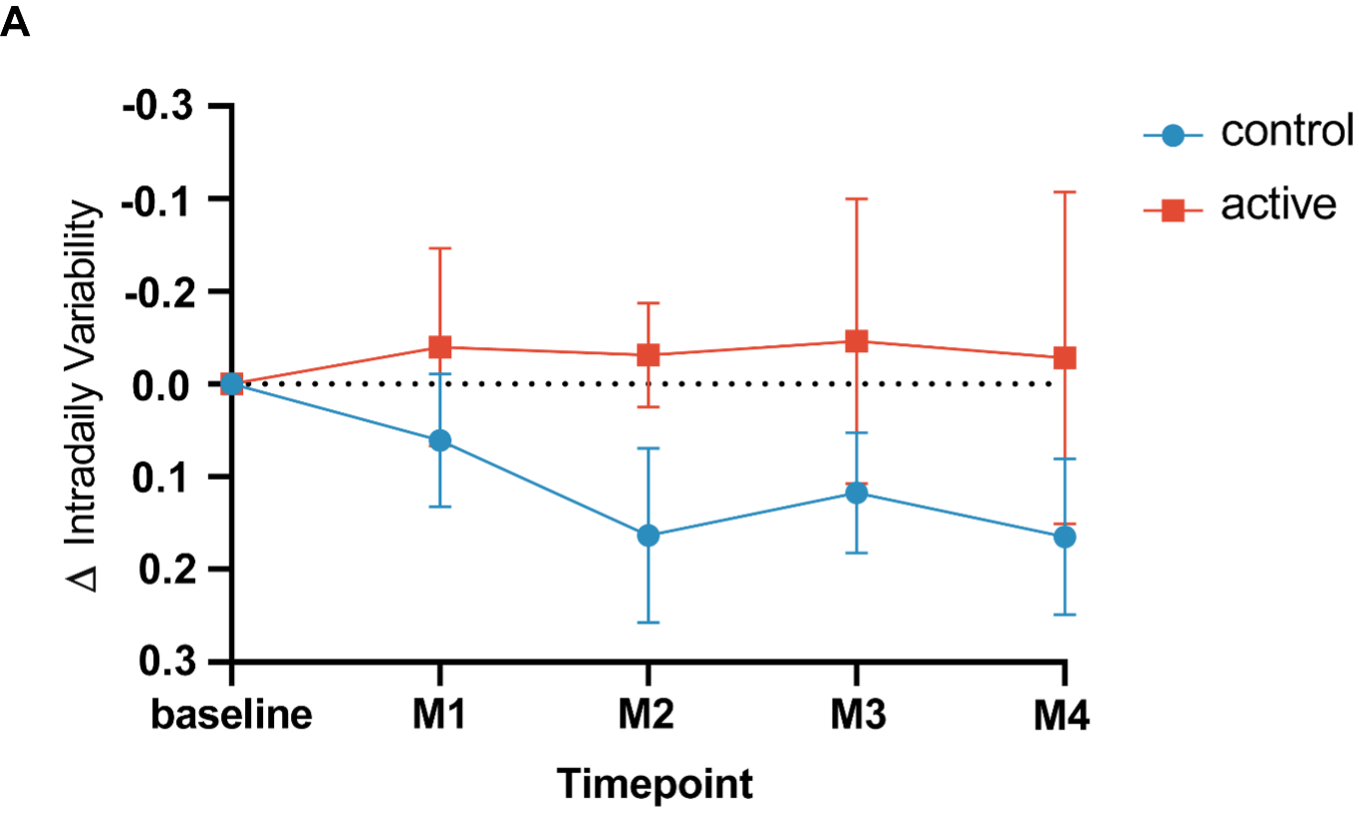

Supplement: S7 Fig — Change in Intradaily Variability demonstrates a trend towards stabilization in the active group but no change in control group. Statistical analysis with a 2-Way ANOVA with Šídák’s multiple comparisons at each timepoint compared to 0 (no-change). Lines indicate means ± SEM. Mean of control = 0.13, mean of active = -0.04. Main effect of intervention, p = 0.22, interaction of intervention x time, p = 0.88, M4 multiple comparison, control, p = 0.30, active, p = 0.99. (PNG) [file pone.0278412.s008.png]
