# Supplementary material for: Gamma frequency sensory stimulation in mild probable Alzheimer’s dementia patients: Results of feasibility and pilot studies
Source: PLoS One. 2022 Dec 1;17(12):e0278412. doi: 10.1371/journal.pone.0278412 (PMC9714926; doi:10.1371/journal.pone.0278412)
Supplement: S1 Table — (PDF) [file pone.0278412.s009.pdf]

Study group	Inclusion criteria	Exclusion criteria
Cognitively normal young and older groups	• Participant is between the ages of 18 - 35 and 50 – 100<br>• Participant is willing and able to sign informed consent form	• Participants who are pregnant.<br>• Participants who do not have healthcare.<br>• Participants who are being treated with acetylcholinesterase inhibitors. (donepezil, galantamine, rivastigmine) or N-methyl-D-aspartate (NMDA) receptor antagonists (eg. Memantine) who have been on a stable dose for less than 60 days.<br>• Participants with history of seizure or epilepsy within the past 24 months.<br>• Active treatment with one or more anti-epileptic agent.<br>• Participants who have had a stroke within the past 24 months.<br>• Participants diagnosed with a known diagnosis of migraine headache.<br>• Active treatment with one or more psychiatric agent (e.g. antidepressants, antipsychotics, etc).<br>• Participants who have an active implantable medical device including but not limited to implantable cardioverter defibrillator (ICD), deep brain stimulator (DBS), cardiac pacemaker, and/or sacral nerve stimulator.<br>• Participants who have contraindications for MRI imaging.<br>• Participants who have a life expectancy of less than 2 years.
Mild AD group	• Participant is between the age–s of 50 - 100.<br>• Participant must have mild Alzheimer's disease with a Mini Mental State Exam (MMSE) score of 19 -26.<br>• Participant is willing to sign informed consent document.<br>• If participant is deemed to not have capacity to sign the informed consent, he/she will need a legally authorized representative to provide surrogate consent.	• Subjects who do not have healthcare.<br>• Subjects who are being treated with N-methyl-D-aspartate (NMDA) receptor antagonists (eg. Memantine).<br>• Subjects on medications that lower seizure threshold such as wellbutrin, ciprofloxacin, levofloxacin, etc.<br>• Subjects with history of seizure or epilepsy within the past 24 months.<br>• Subjects with clinically significant suicide risk and/or suicide attempt in the past 1 year.

		• Subjects with behavioral problems such as aggression/agitation/impulsivity that might interfere with their ability to comply with protocol.<br>• Active treatment with one or more anti-epileptic agent.<br>• Subjects who have had a stroke within the past 24 months.<br>• Subjects diagnosed with migraine headache.<br>• Active treatment with one or more psychiatric agent (e.g. antidepressants, antipsychotics, etc).<br>• Subjects who have an active implantable medical device including but not limited to implantable cardioverter defibrillator (ICD), deep brain stimulator (DBS), cardiac pacemaker, and/or sacral nerve stimulator.<br>• Subjects who have profound hearing or visual impairment.<br>• Subjects who have a life expectancy of less than 2 years.<br>• Subjects who are pregnant.

Table S1. Eligibility criteria.
