# Supplementary material for: Gamma frequency sensory stimulation in mild probable Alzheimer’s dementia patients: Results of feasibility and pilot studies
Source: PLoS One. 2022 Dec 1;17(12):e0278412. doi: 10.1371/journal.pone.0278412 (PMC9714926; doi:10.1371/journal.pone.0278412)
Supplement: S2 Table — (PDF) [file pone.0278412.s010.pdf]

	Change in 40Hz Global Coherence from No Stimulation, Median (Range)[a]	40Hz Combined Stimulation vs No Stimulation p Value[b]
**Young, CN (n=13)**	0.34 (0.17 to 0.61)	<0.001
**Older, CN (n=12)**	0.23 (0.09 to 0.41)	<0.001
**Mild AD (n=16)**	0.22 (0.04 to 0.45)	<0.001

Abbreviations: CN, cognitively normal; AD, Alzheimer's disease.

[a] Ranges from -1 to 1, with a more positive value indicating more increase in the 40Hz global coherence and a more negative value indicating more decrease in the 40Hz global coherence with 40Hz stimulation compared to no stimulation.

[b] Wilcoxon's sign rank test.

Table S2. Changes in scalp EEG global coherence at 40Hz with acute 40Hz combined stimulation, related to Figure 1.
