# Supplementary material for: Gamma frequency sensory stimulation in mild probable Alzheimer’s dementia patients: Results of feasibility and pilot studies
Source: PLoS One. 2022 Dec 1;17(12):e0278412. doi: 10.1371/journal.pone.0278412 (PMC9714926; doi:10.1371/journal.pone.0278412)
Supplement: S3 Table — From top to bottom indicating regions showing change in functional connectivity with the posterior hub of the default mode network including the posterior cingulate cortex (PCC) and precuneus, change in control group connectivity between sessions, change in active group connectivity between sessions, and change between groups between sessions, followed by functional connectivity changes for left and bilateral hippocampus. Right hippocampus showed no changes across all comparisons. P < 0.05 FWE-corrected for all clusters, paired T-test. SMG: Supramarginal Gyrus; FG: Frontal Gyrus; AG: Angular Gyrus; LOC: Lateral Occipital Cortex; FP: Frontal Pole; OP: Occipital Pole. (PDF) [file pone.0278412.s011.pdf]

Resting-State Functional Connectivity Changes

	Region	MNI coordinates			#Voxels	Peak T	Pval (size)
		X	Y	Z			
PCC - Ctrl – Ses2>Ses1							
	rSMG post.	52	-40	44	79	3.04	0.000
	rFG mid.	46	32	24	46	2.5	0.000
	lAG	-40	-54	42	31	2.53	0.000
	lLOC sup.	-40	-60	56	22	0.75	0.003
	rFG mid.	46	26	32	17	2.65	0.020
	rFG sup.	4	26	46	16	2.25	0.029
	lFP	-40	34	8	15	3.75	0042
PCC - Active – Ses2>Ses1							
	N/A						
PCC - Active (Ses2>Ses1) > Ctrl (Ses2>Ses1)							
	lFP	-42	34	8	36	7.32	0.031
	rFP	50	40	4	35	7.01	0.032
Left HPC – Ctrl – Ses2>Ses1							
	N/A						
Left HPC – Active – Ses2>Ses1							
	lLOC sup.	-20	-86	24	27	12.64	0.001
	rLOC sup.	28	-86	32	16	17.37	0.006
Left HPC – Active (Ses2>Ses1) > Ctrl (Ses2>Ses1)							
	N/A						
Bil. HPC – Ctrl – Ses2>Ses1							
	N/A						
Bil. HPC – Active – Ses2>Ses1							
	lLOC sup.	-20	-84	22	62	7.51	0.000
	lOP	-32	-92	2	32	6.06	0.016
Bil. – Active (Ses2>Ses1) > Ctrl (Ses2>Ses1)							
	N/A						

Table S3. Resting-State Functional connectivity clusters.
