# Supplementary material for: Gamma frequency sensory stimulation in mild probable Alzheimer’s dementia patients: Results of feasibility and pilot studies
Source: PLoS One. 2022 Dec 1;17(12):e0278412. doi: 10.1371/journal.pone.0278412 (PMC9714926; doi:10.1371/journal.pone.0278412)
Supplement: S4 Table — (T-test, mean (+/-std)); HPC: Hippocampus; MVN: Medial Visual Network; FC: Functional Connectivity; FNA: Face-name association. (PDF) [file pone.0278412.s012.pdf]

	Control	Active	P-value
Left HPC	1.84e-3(+/-3.27e-4)	1.64e-3(+/-1.85e-4)	0.204
Right HPC	2.00e-3(+/-4.02e-4)	1.67e-3(+/-2.63e-4)	0.099
Bil. HPC	3.84e-3(+/-7.14e-4)	3.31e-3(+/-4.32e-4)	0.127
MVN FC	0.09(+/-0.09)	0.06(+/-0.06)	0.588
FNA recall	7.80(+/-1.48)	6.25(+/-1.89)	0.209
Left HPC	1.84e-3(+/-3.27e-4)	1.64e-3(+/-1.85e-4)	0.204
Right HPC	2.00e-3(+/-4.02e-4)	1.67e-3(+/-2.63e-4)	0.099
Bil. HPC	3.84e-3(+/-7.14e-4)	3.31e-3(+/-4.32e-4)	0.127

Table S4. Baseline comparisons between groups for MRI related outcomes.
