# Supplementary material for: Gamma frequency sensory stimulation in mild probable Alzheimer’s dementia patients: Results of feasibility and pilot studies
Source: PLoS One. 2022 Dec 1;17(12):e0278412. doi: 10.1371/journal.pone.0278412 (PMC9714926; doi:10.1371/journal.pone.0278412)
Supplement: S5 Table — p-values generated using paired T-test. (PDF) [file pone.0278412.s013.pdf]

	Change in Hippocampal Volume (%)			
	Median (Range)	25th %ile	75th %ile	Change in Brain Volume (%)
Control (n=6)	-1.94 (4.09)	-2.81	-0.34	0.45
Active (n=7)	-0.87 (6.52)	-1.97	1.41	0.10
	Between group comparison p-value: 0.034			p=0.419
	Change in Ventricular Volume (%)			
	Median (Range)	25th %ile	75th %ile	Change in Cortical Thickness (%)
Control (n=6)	3.84(4.98)	3.31	5.37	0.23
Active (n=7)	1.23(7.08)	-0.226	3.96	0.52
	Between group comparison p-value: 0.024			p=0.547

Table S5. Structural MRI Statistics
