# Supplementary material for: Gamma frequency sensory stimulation in mild probable Alzheimer’s dementia patients: Results of feasibility and pilot studies
Source: PLoS One. 2022 Dec 1;17(12):e0278412. doi: 10.1371/journal.pone.0278412 (PMC9714926; doi:10.1371/journal.pone.0278412)
Supplement: S1 Protocol — (PDF) [file pone.0278412.s014.pdf]

| | | |
|---|---|---|
| | **Massachusetts Institute of Technology**<br>Committee on the Use of<br>Humans as Experimental Subjects | **Protocol #**<br>1712179268 |

## I. BASIC INFORMATION

| **1. Title of Study** | |
|---|---|
| Pilot Study to evaluate feasibility of sensory stimulation with gamma frequency entrainment | |
| **2. Principal Investigator** | |
| Name: Li-Huei Tsai | Building and Room #:46-4235A |
| Title: Professor; Director of the Picower Institute | Email: lhtsai@mit.edu |
| Department: Picower Institute for Learning and Memory, BCS | Phone: 617-324-1660 |
| **3. Anticipated Dates of Research** | |
| Start Date: January 2018 | Completion Date: January 2023 |

## II. STUDY INFORMATION

**Purpose of Study**

Alzheimer Disease (AD) is the most common cause of dementia, accounting for between 60% and 80% of all dementia cases and the sixth leading cause of mortality in the US [1]. AD associated mortality is estimated to exceed that of breast and prostate cancer combined according to the American Alzheimer's Association [1]. AD affects 46 million people worldwide, with its incidence increasing among the population above 65 years and doubling every five to ten years [2,3]. Likewise, the prevalence of the disease increases exponentially with age, rising from 11% among those 65-74, to almost 50% among those 85 or older [2]. Alzheimer disease also can occur in an early onset presentation in young adults, but this only accounts for less than one percent of the cases [4].

In the United States, an estimated of 5 million people over the age of 65 years are affected by AD and it is projected to rise to 13.8 million in the United States and more than 131 million worldwide by 2050 (a proportionate increase of 250%) [1,3,5]. It means that every 20 years the number of people living with dementia will double. Despite the huge healthcare and economic impact of AD, there is still no disease modifying therapeutics available. In fact, the available therapeutics show low efficacy at best in the treatment of cognitive impairment in dementia. Development of a non-invasive medical device that is effective in slowing cognitive impairment is not only revolutionary but also possibly cost- effective.

Information processing in the brain is thought to occur through synchronized neuronal activity in the form of network oscillations. Activity in the 30-100 Hz range is considered gamma-band oscillation and has been reported to be critical for attention, memory formation, and recall. Disruptions of gamma oscillations, particularly in the 30 – 50 Hz range, are reported as a potential early hallmark of Alzheimer's disease. Our lab previously showed a reduction in 40 Hz gamma power in several Alzheimer's mouse models. Using a non-invasive light (LED) flickering at 40 Hz, we were able to show entrainment of 40 Hz gamma oscillations in the visual cortex along with microglia activation and a significant reduction in amyloid load. The purpose of this

study is to determine whether gamma entrainment through non-invasive 40Hz sensory stimulations (ie. visual and/or auditory) is possible in human association cortices as measured by electroencephalogram (EEG). This study will provide critical insight into potential future studies involving non- invasive 40Hz sensory stimulation as a possible therapeutic strategy for Alzheimer's disease.


1. Sosa-Ortiz AL, Acosta-Castillo I, Prince MJ. Epidemiology of Dementias and Alzheimer's Disease. Arch Med Res. 2012 Nov;43(8):600-8. doi: 10.1016/j.arcmed.2012.11.003.
2. Hebert LE, Weuve J, Scherr PA, Evans DA. Alzheimer disease in the United States (2010-2050) estimated using the 2010 Census. Neurology 2013;80(19):1778-83.
3. World Alzheimer Report 2015: The Global Impact of Dementia http://www.alz.co.uk/research/world-report-2015 (Accessed on October 26, 2017]
4. Bateman RJ, Xiong C, Benzinger TLS, et al. Clinical and Biomarker Changes in Dominantly Inherited Alzheimer's Disease. The New England Journal of Medicine. 2012;367(9):795-804. doi:10.1056/NEJMoa1202753.
5. Hebert LE, Weuve J, Scherr PA, Evans DA. Alzheimer disease in the United States (2010–2050) estimated using the 2010 census. Neurology. 2013;80(19):1778-1783. doi:10.1212/WNL.0b013e31828726f5.


## Study Protocol

Subjects will be evaluated in the Martinos Imaging Center at MIT's McGovern Institute. After screening and obtaining informed consent, the subjects will proceed through the following protocol:

All subjects be treated with light flickering at 30 – 50Hz, sound clicks between 30 – 50 Hz and tactile stimulation using vibration between 30 – 50 Hz using the devices described below for up to 1.5 hours. The subject will be asked to watch the light panel or monitor from which the light is flickering listen to the sounds that will be audible throughout the room, or interact with the tactile stimulation (vibration) device.

1. All subjects will undergo cognitive and mental health evaluations. Elderly participant cohorts will be asked questions from the MiniMental State Exam and the Alzheimer's Disease Assessment Scale – Cognitive Subscale test (ADAS-Cog) and the CCAS Schmahmann scale to evaluate their cognition. If in the tactile stimulation cohort, participants will be asked questions from the Montreal Cognitive Assessment (MoCA). If in the Cognitively Normal Control Cohort, participants will be asked questions from the Cambridge Neuropsychological Test Automated Battery for Down syndrome (CAMCOG-CANTAB-DS battery) and the MoCA. Participants might also be asked to fill out the Patient Health Questionnaire (PHQ-9) to screen for depression and the Hamilton Anxiety Rating Scale (HAM-A) to screen for anxiety. These assessments may take 60-90 minutes to fill out with one of our team members.

2. The subject will have their brain waves recorded by electroencephalogram (EEG). This is done by first placing the EEG cap on their heads with EEG electrodes that touch their scalp using a water-soluble gel. The process of putting the EEG cap on takes 15 - 30 minutes to complete.

3. Then, video recording of the subject will start and the subject will be asked to sit quietly with their eyes open for up to 5 minutes as their brain waves are captured by the EEG machine. The subject will be asked to close their eyes for up to 5 minutes to allow for the EEG to record with eyes closed.

4. The subject will then be asked to open their eyes and look at the light source for up to 5 minutes as described above (eg. LED panel, monitor, etc) which will be blocked with a black curtain.

5. The subject will then receive a random auditory and/or visual and/or tactile stimulation at random frequencies for 5 minutes.

6. The subject will then receive up to 60 minutes of combined stimulation modalities (ex. Light and sound, sound and vibration, light and vibration, or light sound and vibration simultaneously)

7. Then, the subject will sit quietly for up to 10 minutes as the EEG finishes recording and the video recording will end at the end of this study.

8. Brief memory testing described above will be repeated immediately after completion of the EEG recording.

Subjects will be awake during the entirety of the study assessments. An attention button or other queue may be used to intermittently assess if subjects have fallen asleep during the stimulation regime. Visual and auditory stimulation devices will be programmed to control for intensity, frequency of operation, and fail-safes in case of subject duress.

*In the event if a subject experiences discomfort, anxiety, seizure, or any other adverse event, treatment will be stopped, and a research assistant will monitor the subject and call MIT medical for assistance.*

We anticipate approximately 1.5 hours total for stimulation sessions and 1.5 hours total for the behavioral portions
