# Supplementary material for: Gamma frequency sensory stimulation in mild probable Alzheimer’s dementia patients: Results of feasibility and pilot studies
Source: PLoS One. 2022 Dec 1;17(12):e0278412. doi: 10.1371/journal.pone.0278412 (PMC9714926; doi:10.1371/journal.pone.0278412)
Supplement: S2 Protocol — (PDF) [file pone.0278412.s015.pdf]

| | | |
|---|---|---|
| | **Massachusetts Institute of Technology**<br>Committee on the Use of<br>Humans as Experimental Subjects | **Protocol #**<br>1902706647 |

## I. BASIC INFORMATION

| **1. Title of Study** | |
|---|---|
| **Acute Treatment of Alzheimer's disease with Gamma Frequency Stimulation** | |
| **2. Principal Investigator** | |
| Name: Li-Huei Tsai | Building and Room #:46-4235A |
| Title: Professor; Director of the Picower Institute | Email: lhtsai@mit.edu |
| Department: Picower Institute for Learning and Memory, BCS | Phone: 617-324-1660 |
| **3. Anticipated Dates of Research** | |
| Start Date: April 2019 | Completion Date: April 2023 |

## II. STUDY INFORMATION

**Purpose of Study**

Alzheimer Disease (AD) is the most common cause of dementia, accounting for between 60% and 80% of all dementia cases and the sixth leading cause of mortality in the US [1]. AD associated mortality is estimated to exceed that of breast and prostate cancer combined according to the American Alzheimer's Association [1]. AD affects 46 million people worldwide, with its incidence increasing among the population above 65 years and doubling every five to ten years [2,3]. Likewise, the prevalence of the disease increases exponentially with age, rising from 11% among those 65-74, to almost 50% among those 85 or older [2]. Alzheimer disease also can occur in an early onset presentation in young adults, but this only accounts for less than one percent of the cases [4].

In the United States, an estimated of 5 million people over the age of 65 years are affected by AD and it is projected to rise to 13.8 million in the United States and more than 131 million worldwide by 2050 (a proportionate increase of 250%) [1,3,5]. It means that every 20 years the number of people living with dementia will double. Despite the huge healthcare and economic impact of AD, there is still no disease modifying therapeutics available. In fact, the available therapeutics show low efficacy at best in the treatment of cognitive impairment in dementia. Development of a non-invasive medical device that is effective in slowing cognitive impairment is not only revolutionary but also possibly cost- effective.

Information processing in the brain is thought to occur through synchronized neuronal activity in the form of network oscillations. Activity in the 30-100 Hz range is considered gamma-band oscillation and has been reported to be critical for attention, memory formation, and recall. Disruptions of gamma oscillations, particularly in the 30 – 50 Hz range, are reported as a potential early hallmark of Alzheimer's disease. Our lab previously showed a reduction in 40 Hz gamma power in several Alzheimer's mouse models. Using a non-invasive light (LED) flickering at 40 Hz, we were able to show entrainment of 40 Hz gamma oscillations in the visual cortex along with microglia activation and a

significant reduction in amyloid load.

In our initial pilot study (IRB#1712179268), we found that our light and sound device is safe to use in cognitively normal adults and stimulation at 40Hz can be used to entrain their brain activity. During the pilot study, it was determined that 100% of healthy older adults (55 – 75 years old) entrained to visual stimulation, 72% entrained to auditory stimulation, and 100% entrained to a combination of light and sound stimulation. During these trials, subjects reported positive effects including that they felt calmer, more energetic and that the device was easy to use over a two-week period. No negative effects including headaches, seizures or changes in hearing and vision or unexpected side effects were reported from procedures or prolonged stimulation. In anticipation that subjects my experience mild eyestrain, subtitle size was increased on the tablet fixed to the device to aid in ease of reading the subtitles presented during the videos in this study.

The purpose of this next study is to determine whether gamma entrainment through non-invasive 40Hz sensory stimulation is possible in association cortices of subjects with mild AD as measured by electroencephalogram (EEG) during an acute stimulation session. Additionally, we aim to identify the percent of the study population who successfully entrain to each stimulation condition. This study will provide critical insight into potential longitudinal studies involving non-invasive 40Hz sensory stimulation in subject with AD as a possible therapeutic strategy for AD.


1. Sosa-Ortiz AL, Acosta-Castillo I, Prince MJ. Epidemiology of Dementias and Alzheimer's Disease. Arch Med Res. 2012 Nov;43(8):600-8. doi: 10.1016/j.arcmed.2012.11.003.

2. Hebert LE, Weuve J, Scherr PA, Evans DA. Alzheimer disease in the United States (2010-2050) estimated using the 2010 Census. Neurology 2013;80(19):1778-83.

3. World Alzheimer Report 2015: The Global Impact of Dementia http://www.alz.co.uk/research/world-report-2015 (Accessed on October 26, 2017]

4. Bateman RJ, Xiong C, Benzinger TLS, et al. Clinical and Biomarker Changes in Dominantly Inherited Alzheimer's Disease. The New England Journal of Medicine. 2012;367(9):795-804. doi:10.1056/NEJMoa1202753.

5. Hebert LE, Weuve J, Scherr PA, Evans DA. Alzheimer disease in the United States (2010–2050) estimated using the 2010 census. Neurology. 2013;80(19):1778-1783. doi:10.1212/WNL.0b013e31828726f5.


## Experimental Procedures

Prior to the arrival at MIT for this study, each subject will be sent a copy of the informed consent document and the research coordinator will go over inclusion and exclusion criteria by phone. All recruited subjects will be required to bring a family member or a legally authorized representative to their visit and be present during the consenting process.

Subjects will be evaluated in the Martinos Imaging Center at MIT's McGovern Institute. Before initiating any study procedures, each subject will also be screened to determine whether he/she has the capacity to consent. To assess capacity, a board certified MD will ask some questions to evaluate the patient's decision-making ability. In brief, we will assess understanding, the patient's choice, their appreciation of how this trial applies to the patient, and their ability to compare and infer the consequences of their participation in our study. To assess understanding, we will ask, "Can you tell me in your own words what I just told you about our trial using light and sound to treat dementia?". To see if they have the ability to make a decision, "Based on what we just discussed about the trial, what do you choose?". To see if the patient understands how this applies to him/her, "Regardless of what your choice is, can you tell me how you think this trial may benefit or harm you?". And to understand the patient's ability to reason, "How will this trial affect your daily life?". The MD will pay attention to the logical

consistency of the choice based on the reasoning provided. Patients with MMSE > 24 usually have capacity to make medical and health decisions but will be evaluated by the MD as described above. If the subject has capacity, he/she will sign the consent form. If the subject does not have capacity, then both the subject and the caretaker will be required to sign the consent form.

Before their arrival, all subjects will be randomized into one of two groups in a 1:1 ratio for the 60-minute stimulation session described below (step 6). Group 1 will receive 60 minutes of the treatment: light flickering at 30 – 50Hz and sound between 30 – 50 Hz. Group 2 will receive 60 minutes of the control: constant light and white noise. Subjects will not be informed which group they have been randomized to.

1. All subjects will undergo cognitive and mental health evaluations. They may be asked questions from the Mini-Mental State Exam (MMSE), the Alzheimer's Disease Assessment Scale – Cognitive Subscale test (ADAS-Cog), and/or the CCAS Schmahmann scale to evaluate their cognition. They will also complete modified forms from the National Alzheimer's Coordinating Center Uniform Data set. This set includes basic demographic and health questionnaires, as well as the Functional Assessment Scale (FAS), Geriatric Depression Scale (GDS) and the Clinical Dementia Rating (CDR). These questionnaires are attached for your review. These assessments may take 60-90 minutes to fill out with one of our team members. Additional memory tests may also be administered.
2. Subjects will then have their brain waves recorded by electroencephalogram (EEG). This is done by first placing the EEG cap on their heads with EEG electrodes that touch their scalp using a water-soluble gel. The process of putting the EEG cap on takes 15 -30 minutes to complete.
3. Then, video recording of the subject will start and the subject will be asked to sit quietly with their eyes open for 2 minutes as their brain waves are captured by the EEG machine. The subject will then be asked to close their eyes for another 2 minutes to allow for the EEG to record their brain activity with eyes closed.
4. All subjects will receive various combinations of visual, auditory and tactile stimulation, which includes stationary (e.g., constant light, white noise and/or no touch with no vibration) and non-stationary (e.g., a. visual: light flickering at 0.1 - 4Hz and 30 50Hz, b. auditory: sound clicks between 0.1 4 Hz and 30 -- 50 Hz and c. tactile: vibrations between 0.1- 4 Hz and 30 -- 50 Hz) using the devices described below for up to 1.5 hours. The subject will be asked to watch the light panel or monitor from which the light is flickering, listen to the sounds that will be audible throughout the room, or interact with the tactile stimulation (vibration) device. The subject will be asked to either keep their eyes open or closed for short periods. The characteristics of the stimulation other than the frequency (e.g., intensity, duration, addition of a tablet device for entertainment) may also be altered. The upper limit of light intensity used will be 800 lux and the longest duration of treatment time is 60 minutes. Each stimulation period will last 1 5 minutes and be preceded by a baseline period during which the light is obscured and the sound volume is turned off. The order in which each of these stimulation conditions is presented will be randomized. The last stimulation condition will be followed by a baseline period in which the light is obscured and the sound volume is turned off.The characteristics of the stimulation other than the frequency (e.g., intensity, duration, addition of a tablet device for entertainment) may also be altered. The upper limit of light intensity used will be 800 lux and the longest duration of treatment time is 60 minutes. Each stimulation period will last 1 – 5 minutes and be preceded by a baseline period during which the light is obscured and the sound volume is turned off. The order in which each of these stimulation conditions is presented will be randomized. The last stimulation condition will be followed by a baseline period in which the light is obscured and the

sound volume is turned off.

5. The subject will then complete one final 60-minute stimulation session using either the nonstationary bimodal (i.e., simultaneous 30 – 50 Hz auditory and 30 – 50 Hz visual) or the stationary bimodal (i.e., simultaneous white noise and constant light) stimulation depending on their randomization assignment. During the 60 minutes, the subject will be instructed to keep their eyes open and watch a tablet that will be attached onto our device and playing a video clip or a slideshow of photos. Cognitive, mental, and memory evaluations from 1 above may be repeated at the conclusion of the stimulation.

   Subjects will be awake during the entirety of the study assessments. An attention button or other queue may be used to intermittently assess if subjects have fallen asleep during the stimulation regime. Visual and auditory stimulation devices will be programmed to control for intensity, frequency of operation, and fail-safes in case of subject duress.

   We anticipate approximately 1.5 hours total for the behavioral portions (i.e., cognitive and mental health evaluations) and 2 hours total for the stimulation + EEG sessions for each subject.

   *A board certified MD will be present during the study procedures. In the event a subject experiences discomfort, anxiety, seizure, or any other adverse event, the following protocol will be followed:*

1. *The stimulation or testing will be stopped immediately.*
2. *A research assistant will immediately call Emergency Medical Services (EMS) from a MIT phone by dialing 100 or from a cellphone by dialing 617-253-121*
3. *A trained member of the team will monitor the subject and if deemed necessary administer basic life support and/ or obtain vital signs including blood pressure, heart rate and pulse oximetry until emergency staff arrive.*
4. *A trained responder will arrive at the scene to determine if MIT medical is an appropriate venue for the subject to receive care or if the subject should be transported to a hospital.*
5. *Once the subject's safety has been insured, investigators will complete the Adverse Event/Unanticipated Problem Reporting Form. If the event is severe or unanticipated, this form will be submitted within 48 hours of the event occurrence. All other adverse events will be submitted within 10 working days.*
6. *In the event of an adverse reaction, the team certified MD would make the decision along with the subject and their designated family member or legal representative if the study will be safe to continue. If the event was considered serious or unexpected and continuing research protocol may pose a danger to their heath, it is possible that they will be removed from the study for their safety.*

***COVID-19 Cohort:***

Due to the halt of in-person human subjects research, subjects will be offered to complete partial virtual visits instead of completing 1 full in-person visit.

1. Consent: Prior to scheduling a virtual visit, subjects will be sent our consent form for review via MIT DocuSign. Subjects and co-participants will be given the option to discuss the research with

a team member who will be available by telephone, email, ZOOM Meeting, or messaging platform prior to signing and throughout their participation in the study. A finalized copy of the consent form with signatures will be provided to the participant via email or postal service.

2. Virtual Visits: After completion of consent, subjects will be asked to complete a virtual assessment with a study MD or licensed neuropsychologist. Prior to the visit, the subject will be sent the demographic and intake forms via secure redcap survey link. These forms include approved demographic forms A1-5 and new subject intake form. This virtual visit will be conducted over ZOOM and include cognitive testing, and mental health evaluation.

3. In-Person EEG visit: After the completion of their visit, the participant and co-participant will be informed if they meet the inclusion criteria. If they agree and when safe to do so by MIT and Massachusetts recommendations, they will be invited to come to MIT campus (Bldg. 46 Martinos Imaging Center) to complete their EEG session. If they do not meet the criteria, they will not be invited back for an EEG however, they will still be compensated for their time at a rate of $20 per hour for their cognitive testing.
