# Supplementary material for: Gamma frequency sensory stimulation in mild probable Alzheimer’s dementia patients: Results of feasibility and pilot studies
Source: PLoS One. 2022 Dec 1;17(12):e0278412. doi: 10.1371/journal.pone.0278412 (PMC9714926; doi:10.1371/journal.pone.0278412)
Supplement: S3 Protocol — (PDF) [file pone.0278412.s016.pdf]

	**Massachusetts Institute of Technology**<br>Committee on the Use of<br>Humans as Experimental Subjects	**Protocol #**<br>1903763319

I. BASIC INFORMATION

**1. Title of Study**	
**Chronic Treatment of Alzheimer's disease with Gamma Frequency Stimulation**	
**2. Principal Investigator**	
Name: Li-Huei Tsai	Building and Room #:46-4235A
Title: Professor; Director of the Picower Institute	Email: lhtsai@mit.edu
Department: Picower Institute for Learning and Memory, BCS	Phone: 617-324-1660
**3. Anticipated Dates of Research**	
Start Date: April, 2019	Completion Date: April, 2025

II. STUDY INFORMATION

**Purpose of Study**
Alzheimer Disease (AD) is the most common cause of dementia, accounting for 60% to 80% of all dementia cases, and the sixth leading cause of mortality in the US, increasing its deaths by 145% between 2000 and 2017 [1]. AD affects 50 million people worldwide, with its incidence increasing among the population above 65 of age [2,3]. The percentage of people affected by age are 3% among those of 65-74 years to 32% of people age 85 or older [2]. In the United States, an estimated of 5.8 million people over the age of 65 years are affected by AD and it is projected to rise to 13.8 million in the United States and more than 152 million worldwide by 2050 [1,2]. Despite the huge healthcare and economic impact of AD, there are still no disease modifying therapeutics available. In fact, the available therapeutics show low efficacy at best in the treatment of cognitive impairment in dementia. Development of a non-invasive medical device that is effective in slowing cognitive impairment is not only revolutionary but also possibly cost-effective. Information processing in the brain is thought to occur through synchronized neuronal activity in the form of network oscillations. Activity in the 30-100 Hz range is considered gamma-band oscillation and has been reported to be critical for attention, memory formation, and recall. Disruptions of gamma oscillations, particularly in the 30 – 50 Hz range, are reported as a potential early hallmark of Alzheimer's disease. Our lab previously showed a reduction in 40 Hz gamma power in several Alzheimer's mouse models. Using a non-invasive light (LED) flickering at 40 Hz, we were able to show entrainment of 40 Hz gamma oscillations in the visual cortex along with microglia activation and a significant reduction in amyloid load. During our previous trials in healthy subjects, subjects reported positive effects including that they felt calmer, more energetic and that the device was easy to use over a two-week period. No negative effects including headaches, seizures or changes in hearing and vision or unexpected side effects were reported from procedures or prolonged stimulation. The purpose of this study is to determine whether gamma entrainment through non-invasive 40Hz sensory stimulation is possible in association cortices of subjects with mild AD as measured by electroencephalogram (EEG), whether functional connectivity in their brain will change after 6 months of daily treatment with our light and sound device, and whether molecular biomarkers for AD (including RNA sequencing data) change in these subjects as a result of this treatment (RNA sequencing is included based on previous transcriptomic analysis of peripheral leukocytes that showed that inflammation-related genes are related to neurodegenerative disease such as AD and Parkinson's disease [4]). We will also assess cognition, daily activity, and sleep before and after treatment with our light and sound device. Our goals are threefold: 1. To evaluate safety and compliance during the course of at-home use of our device in the AD population; 2. To identify the % of individuals who entrain to the stimulation in our study population; and 3. To asses any cognitive changes during the course of a 6-month treatment with our device. This study will provide critical insight into extended therapy involving non-invasive 40Hz sensory stimulation as a possible therapeutic strategy for mild to moderate

Alzheimer's disease. 1.Alzheimer's Association. 2019 Alzheimer's disease facts and figures. Alzheimers Dement.2019;15(3):321-87. https://doi.org/10.1016/j.jalz.2019.01.010.2.Hebert LE, Weuve J, Scherr PA, Evans DA. Alzheimer disease in the United States (2010-2050)estimated using the 2010 Census. Neurology 2013;80(19):1778-83.3.World Alzheimer Report 2018 - The state of the art of dementia research: New frontiers. NEWFRONTIERS 48 4.Delvaux E, Mastroeni D, Nolz J, Chow N, Sabbagh M, Caselli RJ, Reiman EM, Marshall FJ,Coleman PD. Multivariate analyses of peripheral blood leukocyte transcripts distinguishAlzheimer's, Parkinson's, control, and those at risk for developing Alzheimer's. Neurobiol Aging.2017 Oct; 58:225-237. doi: 10.1016/j.neurobiolaging.2017.05.012. Epub 2017 Jun 20. PubMedPMID: 28716532.2.

Experimental Procedures

Before their arrival, subjects will be screened by telephone by a member of our research team for inclusion/exclusion criteria. Eligible subjects will be randomized into one of two study conditions in a 1:1 ratio. Arm 1 (treatment): subjects will be treated with light flickering at 30 – 50Hz and sound between 30 – 50 Hz; Arm 2 (Placebo): subjects will be treated with constant light and white noise.

Subjects will be evaluated in the room 1161, 46 bldg. at MIT. Before initiating any study procedures, each subject will need to show a picture ID to verify subject's identity. Also, each subject will be screened to determine whether he/she has the capacity to consent. To assess capacity, a board certified MD will ask some questions to evaluate the patient's decision-making ability. In brief, we will assess understanding, the patient's choice, their appreciation of how this trial applies to the patient, and their ability to compare and infer the consequences of their participation in our study. To assess understanding, we will ask, "Can you tell me in your own words what I just told you about our trial using light and sound to treat dementia?". To see if they have the ability to make a decision, "Based on what we just discussed about the trial, what do you choose?". To see if the patient understands how this applies to him/her, "Regardless of what your choice is, can you tell me how you think this trial may benefit or harm you?". And to understand the patient's ability to reason, "How will this trial affect your daily life?". The MD will pay attention to the logical consistency of the choice based on the reasoning provided. Patients with MMSE > 24 usually have capacity to make medical and health decisions but will be evaluated by the MD as described above. Even if the subject is determined to have capacity to sign, both the subject and the caretaker will be required to sign the consent form.

At Visit 1**, a team member will meet the subject at the McGovern Institute located at building 46-1171 (550 Main Street, Cambridge, MA 02139). The study procedures that involve subjects' in-person visits will take place at this building. We will obtain informed consent as described above prior to proceeding with the following procedures. We will collect subject's blood for analyzing biological material before and after the 6-month treatment with our light and sound device. The first blood sample (up to 20 ml) will be obtained during Visit 1, prior to starting the treatment, and the second blood sample (up to 20 ml) will be obtained at the end of Month 6, during Visit 2. We anticipate 30 minutes for collection of the blood samples.

We will do RNA isolation from the blood sample using a commercially available purification kit in room 1161. The complete procedure is described below:

1. Before every phlebotomy visit, we will take the necessary clinical equipment from room 1150-B where it will be stored when it is not being used. We will assemble the equipment in room 1161 following the Standard Operating Procedure named "Human Phlebotomy at a non-healthcare location" approved by EHS and CRC authorities. It describes the proper assembly, how to handle blood spills, and the transportation of specimens to the laboratory. At the end of each phlebotomy

procedure, we will disinfect the clinical equipment and return it to room 1150-B for storage.

2. After we meet with the subject, we will go to room 1161 inside the Martino's Imaging Center to obtain informed consent. In the same room, a certified nurse practitioner will draw their blood.

3. Before the blood draw, the nurse will interview each subject and again obtain informed consent for the blood draw. The subject's blood pressure and weight will be recorded (the subject should weigh at least 110 pounds /50 kg). Then, if the subject agrees to phlebotomy, the subject will sit comfortably in a phlebotomy chair with his/her arm positioned on a padded surface. The nurse will practice universal precautions when performing phlebotomy and handling the blood sample. She will assemble all the materials for the blood draw including the tourniquet, single-use non-sterile gloves, skin disinfectant (e.g. 70% alcohol swabs), syringe and needle or an evacuated tube system with needle and adapter, blood collection tubes, gauze and hypoallergenic tape or bandage.

4. After cleaning the area with skin disinfectant, the nurse will draw up to 30ml of blood into collection tubes. Pressure and a bandage will be applied to the site for hemostasis.

5. RNA will be extracted from the blood samples using a purification kit protocol. The genetic samples will be carried out to the Tsai Laboratory in an insulated and leak-proof transporter for storage. Later, we will ship the samples to BioMicro Center at building 68-322 for RNA sequencing.

   Following phlebotomy, the subject will continue with the following procedures:

6. All subjects will undergo cognitive and mental health evaluations. They will be asked to complete questions from the Mini-Mental State Exam (MMSE), the Alzheimer's Disease Assessment Scale – Cognitive Subscale test (ADAS-Cog), The Montreal Cognitive Assessment (MOCA), and the CCAS Schmahmann scale to evaluate their cognition. They will also complete the National Alzheimer's Coordinating Center Uniform Data set. This set includes basic demographic and health questionnaires, as well as the Functional Assessment Scale (FAS), Geriatric Depression Scale (GDS) and the Clinical Dementia Rating (CDR). These questionnaires are attached for your review. These assessments may take 60-90 minutes to fill out with one of our team members. Additional, memory tests may also be administered.

7. All subjects will undergo brief hearing and vision tests: For the hearing test, we will use an audiometer. The subject will answer a history questionnaire (the "audiometer test form"). We will help the subject put the audiometry headphones on. Then we will present to the subject different tones alternating between frequencies from 250 to 4000 Hz and at an intensity level of 30dB. We will start with the subject's better ear as indicated by their answer in the history questionnaire. If there is no better ear, we will start with their right ear. The visual acuity test will be performed with a six feet distance Snellen Chart. The chart is placed 6ft away from the subject. The evaluation starts with the right eye while covering the left eye. The subject must read lines of letters that go from top to bottom that decrease in size. The process is repeated for the left eye.

   After these initial evaluations, a team member will escort the subject to room 1150 to perform functional structural MRI scans. The scans will be conducted before and after the 6-month treatment regimen, at Month 0 and Month 6 respectively. The procedure will be as follows:

8. The subject will again be evaluated with the MRI screening tool to assess for MRI safety. Each

question answered "yes" will require additional details for safety evaluation. If the participant is found incompatible, the scan will not be performed. If the participant is cleared, they will be asked to change into a linen shirt, pants, remove all jewelry, and complete a screen for additional ferrous metals.

9. The scan will be conducted in a 3T Siemens Magnetom 60cm MRI scanner and will include T1 and SWI sequences. No contrast agents will be used. Set up takes approximately 15 minutes and scanning will take no more than 1 hour. We will analyze structural and functional data using multiple toolbox software, some developed by the Gabrieli Lab at MIT to see changes in the brain over time. During the MRI scan, we will also conduct an fMRI cognitive paradigm which is the face-word recall task. The task consists of 6 runs presenting face-name pairs. It assesses functional activity of working memory by associative encoding. Subjects will press a button to indicate if they think the name corresponds to the face appearing. We will alternate novel and repeated face-name pairs in every run. We will use a check form to track consistency of the task throughout the study by our designed fMRI Scanner run sheet (attached). Additionally, after exiting the MRI, the subject will be asked to complete a memory test of the face-name pairs that appeared during the scan.

   After the MRI scanning, the subject will be directed to room 1159 to be recorded with an EEG system while completing a stimulation session.

10. Subjects will have their brain waves recorded by electroencephalogram (EEG). This is done by first placing the EEG cap on their heads with EEG electrodes that touch their scalp using a water-soluble gel. The process of putting the EEG cap on takes 15 -30 minutes to complete.

11. The subject will be asked to sit quietly with their eyes open as their brain waves are captured by the EEG machine. The subject will then be asked to close their eyes to allow the EEG to record their brain activity with eyes closed.

12. Picture recording of the subject will start, and subjects from both arms will receive various combinations of visual and auditory stimulation, which will include non-stationary (i.e., 30 – 50 Hz) stimulation condition and stationary (e.g., constant light and/or white noise) stimulation condition. The characteristics of the stimulation other than the frequency (e.g., intensity, duration, addition of a tablet device for entertainment) may also be altered. The upper limit of light intensity used will be 800 lux and the longest duration of treatment time is 60 minutes. Each stimulation period will last 1 – 5 minutes and be preceded by a baseline period during which the light is obscured and the sound volume is turned off. The order in which each of these stimulation conditions is presented will be randomized. The last stimulation condition will be followed by a baseline period in which the light is obscured and the sound volume is turned off.

13. The subject will complete one final stimulation session up to 60 minutes using either the non-stationary bimodal (i.e., simultaneous 30 – 50 Hz auditory and 30 – 50 Hz visual) or the stationary bimodal (i.e., simultaneous white noise and constant light) stimulation depending on their randomization assignment. During the stimulation, the subject will be instructed to keep their eyes open and watch a tablet that will be attached onto our device and playing a video clip or a slideshow of photos. Cognitive, mental, and memory evaluations from 6 above may be repeated at the conclusion of the stimulation.

Subjects will be awake during the entirety of the study assessments. An attention button or other queue may be used to assess if subjects have fallen asleep during the stimulation regime. Visual and auditory stimulation devices will be programmed to control for intensity, frequency of operation, and fail-safes in case of subject duress.

Within 72 hours of Visit 1, two members of our team will install our device in the subject's home. Their device will be programmed to administer the light and sound consistent with their arm randomization (treatment or placebo). The light and sound device will also have an affixed camera that only turns on during the stimulation sessions to record the session through a series of pictures taken at short intervals during the stimulation session. The subject will consent for video recording of their session in our consent form. This camera can be obscured when not in use. The daily procedure is explained below:

1. The subject will be asked to turn on our light and sound device, and watch a video playing on the affixed tablet quietly for a total of 30 – 60 minutes daily while comfortably seated 5 feet away from the panel.

2. After the 30 – 60 min session, the subject will be instructed to turn off the device until the following session time and complete a short daily survey for assessing alertness, mood, and daily activities over the past 24 hours. For example, questions may include fatigue levels and caffeine consumption. Through this questionnaire, we will evaluate factors that may vary daily and affect the efficacy of the stimulation as well as document how they felt during the session.

3. Once the session is completed, the subject may go about their normal daily actives.

4. Subjects will be asked to repeat steps 1-3 above daily for a total of 6 months.

After 1 month of daily stimulation sessions at home, the subject will be asked to return to MIT for a brief visit 2. At this visit, we will reset the camera, extract the video recordings and download data from the panel to evaluate for compliance of usage. Cognitive, mental, and memory evaluations performed during Visit 1 may be repeated during this visit. This visit will last approximately 1-2 hours total.

After 3 months of daily stimulation sessions, the subject will be asked to return to MIT for Visit 3. Cognitive, mental, and memory evaluations performed during Visit 1 may be repeated during this visit. In addition, subject will complete a brief EEG using our GENUS device within the parameters listed above for visit 1. This EEG will take no more than 1.5 hours total including setup. Finally, the subject will complete a short MRI lasting approximately 1 hour including setup.

After 6 months of daily stimulation sessions, the subject will be asked to return to MIT for Visit 4 for a repeat of tests that were conducted during Visit 1.

During Visit 4, the subject will complete the activities of Visit 1 following the protocols described above and also repeated below:

1. We will meet the subject at the McGovern Institute located at building 46-1171 and we will go to room 1161 where a certified nurse practitioner will draw their blood.

2. The subject will then have their brainwaves recorded by EEG in room 1159. During the recording, the subjects from both arms will receive various combinations of visual and auditory

stimulation, which will include non-stationary (i.e., 30 – 50 Hz) stimulation condition and stationary (e.g., constant light and/or white noise) stimulation condition. The characteristics of the stimulation other than the frequency (e.g., intensity, duration, addition of a tablet device for entertainment) may also be altered.

3. The subject will be asked to complete one final 60-minute stimulation session using either the treatment condition or the placebo condition depending on their study arm.

4. The subject will undergo brief hearing and vision tests.

5. The subject will undergo cognitive and mental health evaluations. Additional memory tests may also be administered.

6. The subject will again be evaluated with the MRI screening tool to assess for MRI safety and complete both structural and functional MRI scans.

COVID-19 human subjects research suspension response:

Due to the suspension of all in-person humans subject research across MIT as well as for the safety of our at-risk participants, the study schedule was slightly modified during the pandemic. For all participates who did not complete this 6-month visit (visit 4), their visit will be postponed to 9 months if they choose to provide informed re-consent. Subjects who HAVE completed this 6-month visit will be instructed to continue the approved protocol and return at Month 12 for an evaluation.

Subjects who are unable or feel unsafe returning for in-person visits will be asked to complete a virtual visit with the study M.D. This virtual visit will be conducted over ZOOM and include virtual re-consenting with an updated consent form addressing these changes, cognitive testing, and mental health evaluation by the study M.D. These visits will be recorded for grading and note taking purposes. Subjects will be mailed a packet of necessary materials including a prepared tablet with the zoom application and tablet stand for use prior to their visit. The help of assistant or caregiver will be strongly recommended. Technical phone or video support will be provided if necessary. All materials will be returned after the visit by mail using a prepaid shipping label

After Visit 4, all subjects will be given the option of continuing with home treatment for an additional 6 months. If a subject from the control group (Arm 2) decides to continue, they will start daily at-home treatment with light flickering at 30 – 50Hz and sound between 30 – 50 Hz. If a subject from the treatment group (Arm 1) decides to continue, they will continue with their original stimulation condition. The subjects will receive monthly calls at home to check on how everyone is doing.

If a subject decides NOT to continue treatment for an additional 6 months, the subject will be instructed to stop stimulation sessions after Visit 4. These subjects will be invited to return to MIT for a final visit at Month 7 to complete an additional session of cognitive and mental health evaluations and EEG described above.

If the subject wishes to continue for an additional 6 months (12 months total), the subject will be invited to return to MIT for Visit 5 at the end of 12 months to complete an additional session of cognitive and

mental health evaluations and EEG as described above. MRI imaging may also be performed.

If the subject wishes to stop continuation in the study at this point, they will be instructed to stop stimulation sessions after 12 months of use. These subjects will be invited to return to MIT for a final visit at Month 13 to complete an additional session of cognitive and mental health evaluations and EEG described above.

Long Term Extension:

Participants who complete the study protocol and finishes the final study visit at Month 13 will be offered a long-term extension for a total of 3 years. Participants will be asked to continue daily home stimulation sessions and return yearly to complete an EEG, MRI, and cognitive testing. During this time, we will NOT continue video recording or collecting daily actigraphy from their activity monitors. Subjects will be instructed to follow all the same daily procedures when completing at home stimulation as described above.

***In the event a subject experiences discomfort, anxiety, seizure, or any other adverse event, the stimulation will be stopped immediately. A board-certified MD will be present during the study procedures. In the event a subject experiences discomfort, anxiety, seizure, or any other adverse event, the following protocol will be followed: The stimulation or testing will be stopped immediately and a research assistant will immediately call Emergency Medical Services (EMS) from a MIT phone by dialing 100 or from a cellphone by dialing 617-253-1212. A trained member of the team will monitor the subject and if deemed necessary administer basic life support and/or obtain vital signs including blood pressure, heart rate and pulse oximetry until emergency staff arrive who will determine if MIT medical is an appropriate venue for the subject to receive care or if the subject should be transported to a hospital. Once the subject's safety has been insured, investigators will complete the Adverse Event/Unanticipated Problem Reporting Form. If the event is severe or unanticipated, this form will be submitted within 48 hours of the event occurrence. All other adverse events will be submitted within 10 working days. In the event of an adverse reaction, a board-certified MD would make the decision along with the subject and their designated family member or legal representative if the study will be safe to continue. If the event was considered serious or unexpected and continuing research protocol may pose a danger to their heath, they will be removed from the study for their safety.*

***Throughout the study, members of our research team will remain in contact with the subject to insure there are no negative side effects due to our therapy. After each week, a researcher will call to complete a telephone survey regarding the emergence of any side effects both during stimulation and within the previous week, as well as to hear any comments they have regarding the treatment. Subjects will be provided with our direct contact information (telephone # and email) to contact us if any adverse effects are experienced at home between each monthly checkup. The subjects will be instructed to contact a member of our research team and stop treatment immediately until advised by a member of our team if any negative side effects are experienced. In the event of an emergency, the subject will be instructed to stop treatment immediately and call 911.*
